# Evaluation of Serum Glucose and Kidney Disease Progression Among Patients With Diabetes

**DOI:** 10.1001/jamanetworkopen.2021.27387

**Published:** 2021-09-29

**Authors:** Hae Hyuk Jung

**Affiliations:** 1 Department of Medicine, Kangwon National University Hospital, Kangwon National University School of Medicine, Chuncheon, South Korea

## Abstract

**Importance:**

The appropriate target of glycemic control in diabetic kidney disease is unclear.

**Objective:**

To investigate optimal on-treatment glycemic levels associated with slowing of diabetic kidney disease progression.

**Design, Setting, and Participants:**

This retrospective cohort study was conducted using nationwide Korean cohorts from the National Health Information Database from 2005 to 2019. Included individuals were adults with diabetes using antihyperglycemic agents with and without chronic kidney disease (CKD) identified from participants aged 40 to 74 years in a nationwide health screening survey conducted from 2009 to 2010. Data were analyzed from October 2020 through March 2021.

**Exposure:**

On-treatment fasting blood glucose (FBG) level.

**Main Outcomes and Measures:**

The primary outcome was a composite of doubling of serum creatinine, end-stage kidney disease, or death from CKD.

**Results:**

Among 183 049 adults with diabetes using antihyperglycemic agents (mean [SD] age, 61.7 [8.4] years; 99 110 [54.1%] men), there were 131 401 individuals with dipstick albuminuria or an estimated glomerular filtration rate (eGFR) of 15 to 59 mL/min/1.73 m^2^ (mean [SD] age, 62.4 [8.3] years; 71 280 [54.2%] men) and 51 648 individuals with no CKD (mean [SD] age, 59.6 [8.4] years; 27 830 [53.9%] men). During 9 years of follow-up, the primary outcome occurred among 13 802 individuals with CKD (10.5%) and 1421 individuals with no CKD (2.8%). On-treatment FBG level had a J-shaped hazard ratio (HR) curve for the primary outcome. Among patients with albuminuria, FBG levels of 126 mg/dL to less than 140 mg/dL (HR, 0.87; 95% CI, 0.81-0.94) and 140 mg/dL to less than 160 mg/dL (HR, 0.90; 95% CI, 0.84-0.96) were associated with decreased risk and levels of 160 mg/dL to less than 180 mg/dL were associated with increased risk (HR, 1.10; 95% CI, 1.03-1.18) compared with FBG levels of 110 mg/dL to less than 126 mg/dL. Among patients with decreased eGFR, FBG levels of 80 mg/dL to less than 100 mg/dL (HR, 1.30; 95% CI, 1.20-1.42) and levels of 160 mg/dL to less than 180 mg/dL (HR, 1.13; 95% CI, 1.04-1.23) were associated with increased risk of the primary outcome compared with FBG levels of 110 mg/dL to less than 126 mg/dL. Among patients with no CKD, FBG levels of 80 mg/dL to less than 100 mg/dL (HR, 1.29; 95% CI, 1.01-1.65) and levels of 126 mg/dL to less than 140 mg/dL (HR, 1.23; 95% CI, 1.03-1.47) were associated with increased risk compared with FBG levels of 110 mg/dL to less than 126 mg/dL. Among patients with no albuminuria at baseline, FBG levels of 140 mg/dL to less than 160 mg/dL (HR, 1.14; 95% CI, 1.09-1.20) were associated with increased risk of new-onset albuminuria, while levels of 100 mg/dL to less than 110 mg/dL were not associated with increased risk compared with FBG levels of 110 mg/dL to less than 126 mg/dL. For all-cause mortality, while FBG levels of 160 mg/dL to less than 180 mg/dL (HR, 1.20; 95% CI, 1.12-1.28) were associated with increased risk among patients with albuminuria, FBG levels of 140 mg/dL to less than 160 mg/dL were associated with increased risk among patients with decreased eGFR (HR, 1.10; 95% CI, 1.04-1.16) and those with no CKD (HR, 1.10; 95% CI, 1.00-1.21) compared with levels of 110 mg/dL to less than 126 mg/dL.

**Conclusions and Relevance:**

These findings suggest that intensive vs standard glucose control may not be associated with increased protection for the progression of established diabetic kidney disease and that timely control is important for prevention. However, careful glycemic control would still be associated with improved overall outcomes among patients with CKD, particularly those with no albuminuria.

## Key Points

**Question:**

What is the optimal glycemic target associated with slowing of the progression of diabetic kidney disease?

**Findings:**

In this cohort study of 183 049 adults with diabetes using antihyperglycemic agents, among individuals with albuminuria, a fasting blood glucose level of 126 mg/dL to less than 160 mg/dL was associated with decreased risk of the composite outcome of serum creatinine doubling, end-stage kidney disease, or death from chronic kidney disease. Among individuals with no albuminuria, a fasting blood glucose level of 100 mg/dL to less than 126 mg/dL was associated with decreased risk of the composite outcome and a level of less than 140 mg/dL was associated with decreased risk of new-onset albuminuria.

**Meaning:**

These findings suggest that the optimal on-treatment glucose level associated with slowing the progression of established diabetic kidney disease is higher than the level associated with preventing the development of diabetic kidney disease.

## Teaser

This cohort study examines the association of fasting blood glucose level with risk of kidney disease progression among patients with diabetes.

## Introduction

The prevalence of end-stage kidney disease (ESKD) is rapidly increasing worldwide,^1^ and diabetes is the leading cause of ESKD. Decreasing blood glucose levels among patients with diabetes is associated with decreased incidence of diabetic kidney disease. However, the appropriate blood glucose target associated with slowed progression of diabetic kidney disease is unknown.

In randomized controlled trials that compared different glycemic targets among patients with type 2 diabetes, intensive vs standard control consistently decreased the onset and increase of albuminuria.^2,3,4^ However, trials did not find a clear benefit of intensive control for slowing kidney disease progression. In a 2017 Cochrane review,^5^ intensive control targeting hemoglobin A_1c_ (HbA_1c_) levels less than 7% (to convert to proportion of total hemoglobin, multiply by 0.01) or fasting blood glucose (FBG) level less than 120 mg/dL (to convert to mmol/L, multiply by 0.0555), intensive vs less stringent control was not associated with significant differences in risk of doubling serum creatinine, ESKD, or mortality, whereas it was associated with decreased risk of incident or increasing albuminuria. In contrast to the review that included trials with a mean of approximately 5 years of follow-up, an extended 22-year follow-up study of the Diabetes Control and Complications Trial for type 1 diabetes found that intensive control vs conventional control was associated with decreased incidence of an estimated glomerular filtration rate (eGFR) of less than 60 mL/min/1.73 m^2^.^6^ It is possible that long-term glucose control is associated with slowed kidney disease progression and eventually prevention of progression to ESKD. However, long-term, large-scale studies will be needed to ascertain the kidney benefit of glycemic control, and there is a lack of data on the appropriate treatment target of blood glucose among patients with diabetic kidney disease. To investigate optimal on-treatment glycemic levels associated with slowing the progression of diabetic kidney disease, we estimated risks for doubling serum creatinine, ESKD, and mortality by on-treatment FBG levels and baseline kidney disease status in population-representative groups of patients with diabetes and chronic kidney disease (CKD) in Korea.

## Methods

This cohort study’s design was approved by the institutional review board of Kangwon National University Hospital, which waived the need for informed consent because the data used were deidentified prior to analysis. The study followed the reporting requirements of the Strengthening the Reporting of Observational Studies in Epidemiology (STROBE) reporting guideline for cohort studies.

### Participants

**Figure 1.**
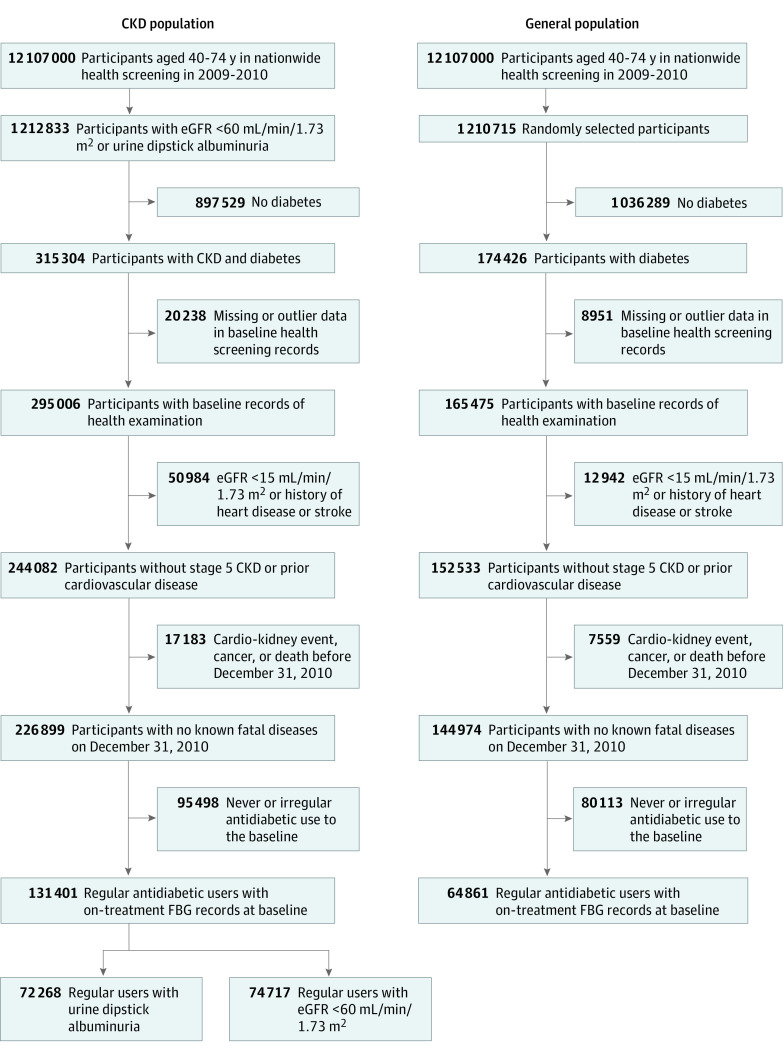
Flowchart of Participant Selection CKD indicates chronic kidney disease; eGFR, estimated glomerular filtration rate; FBG, fasting blood glucose.

This study was conducted in retrospective cohorts generated from the National Health Information Database, a public database for the entire population of Korea maintained by the National Health Insurance Service (NHIS).^7^ Patients with CKD were identified from participants aged 40 to 74 years in the nationwide health screening survey in 2009 or 2010, when serum creatinine was first measured in the survey. From 12.1 million survey participants, 1 212 833 individuals with dipstick albuminuria 1+ at least once or trace at least twice during health examinations from 2007 to 2010 or with an eGFR of less than 60 mL/min/1.73 m^2^ at a health examination in 2009 or 2010 were identified, and their health examination and NHIS reimbursement records from January 1, 2005, to December 31, 2019, were collected. From 1 212 833 patients with CKD, 315 304 individuals who had diabetes (defined as a FBG level ≥ 126 mg/dL or a prescription of antihyperglycemic agents for ≥90 days per year) at baseline (ie, January 1, 2011) were identified. From 315 304 patients with CKD and diabetes, 20 238 individuals with missing or outlier data, 50 984 individuals with a history of heart disease or stroke or an eGFR of less than 15 mL/min/1.73 m^2^, and 17 183 individuals who died or developed cardiovascular diseases, major cancers, or ESKD before baseline were excluded to minimize confounding by prior diseases. Among the remaining 226 899 patients, 131 401 individuals who had initiated glucose-lowering medication before January 1, 2010, and received the medication regularly to December 31, 2010 (ie, prescription for > one-half of the days from medication initiation) were included in the final analysis to evaluate long-term outcomes of on-treatment glycemic levels among patients with CKD and diabetes (Figure 1).

In addition, among 12 107 000 participants in the nationwide health screening survey, 1 210 715 individuals (10.0%) were randomly selected to generate a nationally representative sample. From these selected survey participants, 174 426 individuals who had diabetes at baseline were identified. Among these, 8951 individuals with missing or outlier data, 12 942 individuals with an eGFR of less than 15 mL/min/1.73 m^2^ or a history of heart disease or stroke, and 7559 individuals who died or developed ESKD, cardiovascular diseases, or major cancers before baseline were excluded. Among the remaining 144 974 patients, 64 861 individuals who had initiated glucose-lowering medication before 2010 and received the medication regularly to the baseline were included in the final analysis.

### Blood Glucose and Covariates

Participants underwent medical health examinations generally at 2-year intervals. During the examination, FBG level was measured from samples collected after an 8-hour to 12-hour fast. Because the risk thresholds of hyperglycemia were different depending on antidiabetic use status,^8,9,10^ on-treatment and untreated FBG records were separated. On-treatment FBG level was considered to be the FBG level for a patient who had received antihyperglycemic agents for 90 or more days in the year of measurement (eTable 1 in the Supplement). In each year of follow-up, the mean of FBG values from the year of cohort entry (2005) was calculated and classified into 8 categories (ie, 30 mg/dL to <80 mg/dL, 80 mg/dL to <100 mg/dL, 100 mg/dL to <110 mg/dL, 110 mg/dL to <126 mg/dL, 126 mg/dL to <140 mg/dL, 140 mg/dL to <160 mg/dL, 160 mg/dL to <180 mg/dL, and 180 mg/dL to 900 mg/dL) incorporating cutoffs for prediabetes and diabetes.^11^ In each year, antidiabetic use status was also determined. Use was considered regular if the prescription was for more than one-half of each medication period (from medication initiation to each year of follow-up) and irregular when the prescription was for one-half or less of each period.

With baseline data, the variables of age, sex, albuminuria status, time of antidiabetic initiation, antihypertensive and statin use statuses, systolic blood pressure level, body mass index (BMI; calculated as weight in kilograms divided by height in meters squared), family history of cardiovascular disease, income, smoking status, physical exercise frequency, drinking amounts, and total, high-density lipoprotein (HDL), and low-density lipoprotein (LDL) cholesterol levels were categorized (eAppendix in the Supplement). We calculated eGFR from serum creatinine using the Chronic Kidney Disease Epidemiology Collaboration creatinine equation.^12^

### Outcomes

Study outcomes were identified from January 1, 2011, to December 31, 2019. The primary composite outcome of major kidney events was doubling of serum creatinine, ESKD, or death from CKD. Secondary outcomes included components of the primary outcome, new-onset albuminuria, major cardiovascular events, and all-cause mortality. Doubling of serum creatinine from baseline was identified using biennial health examination records. We identified ESKD as dialysis for 90 or more days per year or kidney transplantation, using information retrieved from NHIS reimbursement records (eTable 1 in the Supplement). New-onset albuminuria was identified as the first occurrence of dipstick albuminuria of 1+ or more among participants with no albuminuria at baseline. Major cardiovascular events were identified as revascularization or critical care unit admission for acute coronary syndrome or acute ischemic stroke or death from cardiovascular diseases. Specific causes of death were determined using death certificates from Statistics Korea. For new-onset albuminuria (or creatinine doubling), censoring occurred with the last measurement of urine albumin (or serum creatinine). For other outcomes, censoring occurred with death or at the end of the study (ie, December 31, 2019).

### Statistical Analysis

Hazard ratios (HRs) were estimated in Cox models with time-varying covariates. The timely mean of on-treatment and untreated FBG levels were entered as time-varying covariates to obtain HRs of on-treatment FBG level adjusting for untreated levels. Yearly updated antidiabetic use status was also entered as a time-varying covariate. The continuous variable of baseline eGFR and the categorized variables of baseline age, sex, albuminuria status, time of antidiabetic initiation, antihypertensive and statin use statuses, systolic blood pressure, BMI, family history of cardiovascular disease, income level, smoking status, exercise frequency, drinking amount, and total, HDL, and LDL cholesterol levels were entered as fixed covariates (eTable 2 in the Supplement). Absolute risks were estimated as adjusted incidence rates per patient-year. Incidence and 95% CI were calculated by multiplying the adjusted HR and its 95% CI by a constant to make the sum of the products of incidence rates and patient-years match the sum of the numbers of observed events in each FBG category. To display associations of continuous FBG values and HRs, spline regression analyses were performed adjusting for the same covariates. The timely mean of on-treatment FBG level was modeled using restricted cubic splines with 5 knots, at percentiles 5, 27.5, 50, 72.5, and 95. In all models, intensively lowered FBG level (ie, 110 mg/dL to <126 mg/dL as a categorized level or 120 mg/dL as a continuous value) was set as the reference.

To estimate population-level risks, analyses were conducted in albuminuria, decreased eGFR, and general populations (ie, individuals with and without CKD who were representative of the general population in Korea). Subgroup analyses stratified by albuminuria or eGFR level were performed to evaluate outcome modification associated with CKD. Analyses stratified by albuminuria level were performed among the albuminuria and general populations and those by eGFR levels were performed among the decreased eGFR and general populations to provide population-representative results. Furthermore, analyses were performed after classifying participants into 1 of 4 groups by the presence or absence of albuminuria and decreased eGFR to evaluate independent and additive outcomes associated with albuminuria and decreased eGFR. In addition, to explore the outcomes associated with transient creatinine increase, the analyses for creatinine doubling were performed after excluding participants whose creatinine doubled from baseline but decreased to less than 2-fold at the last measurement. Analyses including baseline FBG level rather than timely means of levels were also performed to compare outcome risks of baseline and time-updated levels. The proportional hazard assumption in Cox models was tested by including time-dependent interaction terms of on-treatment FBG levels and a log function of follow-up time. Statistical analyses were conducted using SAS statistical software version 9.4 (SAS Institute). Data are presented as numbers and percentages, means and SDs, HRs and 95% CIs, or incidence rates and 95% CIs. *P* values were 2-sided, and *P* < .05 was considered to indicate statistical significance. Given the exploratory nature of this study, no adjustments were made for multiple comparisons. Data were analyzed from October 2020 through March 2021.

## Results

### Characteristics of Study Participants

Among 183 049 participants (mean [SD] age, 61.7 [8.4] years; 99 110 [54.1%] men), including 131 401 individuals with CKD (mean [SD] age, 62.4 [8.3] years; 71 280 [54.2%] men) and 51 648 individuals without CKD (mean [SD] age, 59.6 [8.4] years; 27 830 [53.9%] men), there were 72 268 individuals with dipstick albuminuria, 74 717 individuals with an eGFR of 15 mL/min/1.73 m^2^ to less than 60 mL/min/1.73 m^2^, and 64 861 individuals from the general population (Table). The distribution of follow-up FBG levels was widespread, and the rate of inadequately decreased FBG level was higher in the albuminuria population, while the rate of excessively decreased FBG level was higher in the decreased eGFR population compared with the general population (eFigure 1 in the Supplement).

**Table.** Baseline Characteristics of Study Participants

Characteristic and subgroup	Participants, No. (%) (N = 183 049)		
	CKD with dipstick albuminuria (n = 72 268)	CKD with eGFR<60 mL/min/1.73 m^2^ (n = 74 717)	General population (n = 64 861)
Age, mean (SD), y	59.8 (8.5)	65.3 (7.0)	60.2 (8.5)
Men	46 709 (64.6)	33 842 (45.3)	34 866 (53.8)
Women	25 559 (35.4)	29 995 (46.2)	40 875 (54.7)
10-y cardiovascular risk, mean (SD), %^a^	17.1 (11.6)	18.6 (11.3)	14.5 (10.6)
eGFR, mL/min/1.73 m^2^			
≥120	282 (0.4)	0	245 (0.4)
90 to <120	20 131 (27.9)	0	22 160 (34.2)
60 to <90	36 271 (50.2)	0	34 863 (53.8)
45 to <60	9682 (13.4)	58 994 (79.0)	5988 (9.2)
30 to <45	4135 (5.7)	12 760 (17.1)	1319 (2.0)
15 to <30	1767 (2.4)	2963 (4.0)	286 (0.4)
Albuminuria status			
No albuminuria	0	59 133 (79.1)	57 679 (88.9)
Dipstick albumin 1+ or trace^b^	40 835 (56.5)	6889 (9.2)	4059 (6.3)
Dipstick albumin ≥2+^c^	31 433 (43.5)	8695 (11.6)	3123 (4.8)
Antihyperglycemic agent use^d^			
Metformin	49 526 (68.5)	49 022 (65.6)	44 643 (68.8)
Sulfonylurea	54 315 (75.2)	55 374 (74.1)	47 668 (73.5)
DPP4 inhibitor	2949 (4.1)	2779 (3.7)	2914 (4.5)
Glitazone	5891 (8.2)	6350 (8.5)	6178 (9.5)
Insulin	10 926 (15.1)	10 623 (14.2)	4895 (7.5)
Antidiabetic initiation			
≤2005	47 057 (65.1)	51 270 (68.6)	37 797 (58.3)
2006 or 2007	12 637 (17.5)	12 739 (17.0)	13 317 (20.5)
2008 or 2009	12 574 (17.4)	10 708 (14.3)	13 747 (21.2)
Antihypertensive use			
Never	18 404 (25.5)	14 232 (19.0)	24 089 (37.1)
Irregular	3240 (4.5)	2958 (4.0)	2820 (4.3)
Regular	50 624 (70.1)	57 527 (77.0)	37 952 (58.5)
Statin use			
Never	38 730 (53.6)	38 920 (52.1)	37 212 (57.4)
Irregular	11 334 (15.7)	12 091 (16.2)	9709 (15.0)
Regular	22 204 (30.7)	23 706 (31.7)	17 940 (27.7)
FBG, mean (SD), mg/dL	156.0 (47.6)	140.2 (44.6)	142.9 (41.6)
Systolic BP, mean (SD), mm Hg	132.8 (13.8)	132.0 (13.7)	129.4 (13.1)
Total cholesterol, mean (SD), mg/dL	198.7 (32.2)	196.1 (32.1)	195.1 (30.9)
HDL cholesterol, mean (SD), mg/dL	49.7 (12.2)	49.1 (12.3)	50.5 (12.0)
LDL cholesterol, mean (SD), mg/dL	108.5 (36.2)	108.6 (35.6)	107.5 (33.9)
BMI, mean (SD)	25.4 (3.3)	25.3 (3.1)	25.2 (3.1)
Family history of cardiovascular disease	6549 (9.1)	5763 (7.7)	5856 (9.0)
Income level			
Highest	14 174 (19.6)	16 057 (21.5)	13 331 (20.6)
High	17 968 (24.9)	19 707 (26.4)	16 502 (25.4)
Middle	17 259 (23.9)	17 371 (23.2)	15 679 (24.2)
Low	14 078 (19.5)	13 160 (17.6)	12 177 (18.8)
Lowest	8789 (12.2)	8422 (11.3)	7172 (11.1)
Smoking status			
Never smoked	38 831 (53.7)	52 516 (70.3)	40 183 (62.0)
Former smoker	15 306 (21.2)	12 026 (16.1)	11 609 (17.9)
Current smoker	18 131 (25.1)	10 175 (13.6)	13 069 (20.1)
Physical exercise frequency			
<1 d/wk	16 600 (23.0)	19 748 (26.4)	14 926 (23.0)
1-2 d/wk	14 614 (20.2)	12 703 (17.0)	12 387 (19.1)
3-4 d/wk	17 174 (23.8)	15 958 (21.4)	15 233 (23.5)
≥5 d/wk	23 880 (33.0)	26 308 (35.2)	22 315 (34.4)
Drinking amounts			
None	41 306 (57.2)	55 789 (74.7)	40 883 (63.0)
1-7 drinks/wk	11 044 (15.3)	8795 (11.8)	9356 (14.4)
8-28 drinks/wk	13 773 (19.1)	7591 (10.2)	10 441 (16.1)
≥29 drinks/wk	6145 (8.5)	2542 (3.4)	4181 (6.4)

Abbreviations: BMI, body mass index (calculated as weight in kilograms divided by height in meters squared); BP, blood pressure; CKD, chronic kidney disease; DPP4, dipeptidyl peptidase 4; eGFR, estimated glomerular filtration rate; FBG, fasting blood glucose; HDL, high-density lipoprotein; LDL, low-density lipoprotein.

SI conversion factors: To convert FBG to mmol/L, multiply by 0.0555; total, HDL, and LDL cholesterol to mmol/L, multiply by 0.0259.

^a^ The predicted 10-year risk of cardiovascular disease was calculated using the 2018 revised Pooled Cohort Equations.

^b^ The subgroup had dipstick albuminuria 1+ at least once or trace at least twice during health screenings from 2007 to 2010.

^c^ The subgroup had dipstick albuminuria ≥2+ at least once during health screenings from 2007 to 2010.

^d^ The values are the number of participants who had received each antihyperglycemic agent regularly to the baseline (ie, January 1, 2011).

### Population-Level Analyses

**Figure 2.**
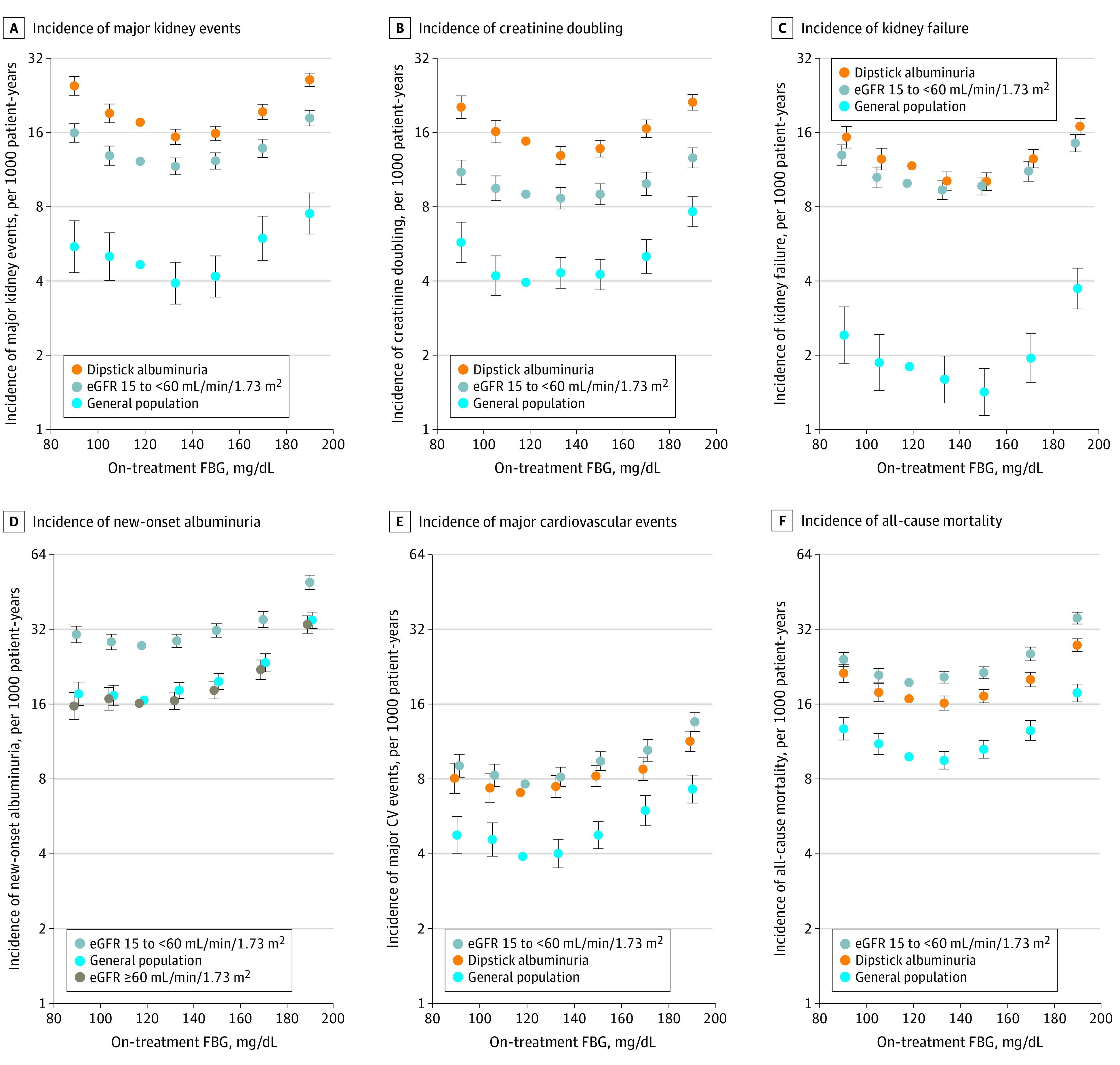
Adjusted Incidence of All Outcomes in Albuminuria, Decreased Estimated Glomerular Filtration Rate (eGFR), and General Populations Incidence rates (log-scale y axis) and 95% CIs (error bars) in fasting blood glucose (FBG) level categories (ie, 80 mg/dL to <100 mg/dL, 100 mg/dL to <110 mg/dL, 110 mg/dL to <126 mg/dL, 126 mg/dL to <140 mg/dL, 140 mg/dL to <160 mg/dL, 160 mg/dL to <180 mg/dL, and 180 mg/dL to 900 mg/dL) were calculated by multiplying adjusted hazard ratios and 95% CIs by a constant to make the sum of the products of incidence rates and patient-years equal the total number of observed events. The rare FBG level of less than 80 mg/dL was excluded from the plots. The on-treatment FBG level of 110 mg/dL to <126 mg/dL was set as the reference. The major kidney event was doubling serum creatinine or kidney failure. Kidney failure was defined as end-stage kidney disease or death from chronic kidney disease. CV indicates cardiovascular.

During 9 years of follow-up, the composite outcome of major kidney events was found in 11 120 individuals (15.4%) in the albuminuria population, 8048 individuals (10.8%) in the decreased eGFR population, and 2787 individuals (4.3%) in the general population (eTable 3 in the Supplement). Overall, on-treatment FBG level had a J-shaped curve for HRs of major kidney events, and the HR was lowest at FBG levels between 110 mg/dL and 160 mg/dL (Figure 2A). Specifically, in the albuminuria population, FBG levels of 126 mg/dL to less than 140 mg/dL (HR, 0.87; 95% CI, 0.81 to 0.94) and 140 mg/dL to less than 160 mg/dL (HR, 0.90; 95% CI, 0.84 to 0.96) were associated with decreased risk of the composite outcome and FBG levels of 160 mg/dL to less than 180 mg/dL (HR, 1.10; 95% CI, 1.03 to 1.18) were associated with increased risk of the composite outcome compared with FBG levels of 110 mg/dL to less than 126 mg/dL. Among patients with decreased eGFR, FBG levels of 80 mg/dL to less than 100 mg/dL (HR, 1.30; 95%, CI 1.20-1.42) and levels of 160 mg/dL to less than 180 mg/dL (HR, 1.13; 95% CI, 1.04-1.23) were associated with increased risk of the primary outcome compared with FBG levels of 110 mg/dL to less than 126 mg/dL (Figure 2A). The J-shaped HR results were also observed for creatinine doubling and kidney failure (Figure 2B and 2C). In contrast, FBG levels had flat and then increasing HR results for new-onset albuminuria, and FBG levels of 140 mg/dL to less than 160 mg/dL (HR, 1.14; 95% CI, 1.09-1.20) were associated with increased risk of new-onset albuminuria, but levels less than 110 mg/dL were not associated with increased risk compared with FBG levels of 110 mg/dL to less than 126 mg/dL (Figure 2D). Meanwhile, FBG levels of 140 mg/dL to less than 160 mg/dL were consistently associated with increased risk for major cardiovascular events in albuminuria, decreased eGFR, and general populations (Figure 2E). As for all-cause mortality, compared with levels of 110 mg/dL to less than 126 mg/dL, FBG levels of 160 mg/dL to less than 180 mg/dL (HR, 1.20; 95% CI, 1.12-1.28) were associated with increased risk among patients with albuminuria, and FBG levels of 140 mg/dL to less than 160 mg/dL (HR, 1.10; 95% CI, 1.04-1.16) were associated with increased risk among patients with decreased eGFR (Figure 2F).

### Stratified Analyses by Kidney Disease Status

**Figure 3.**
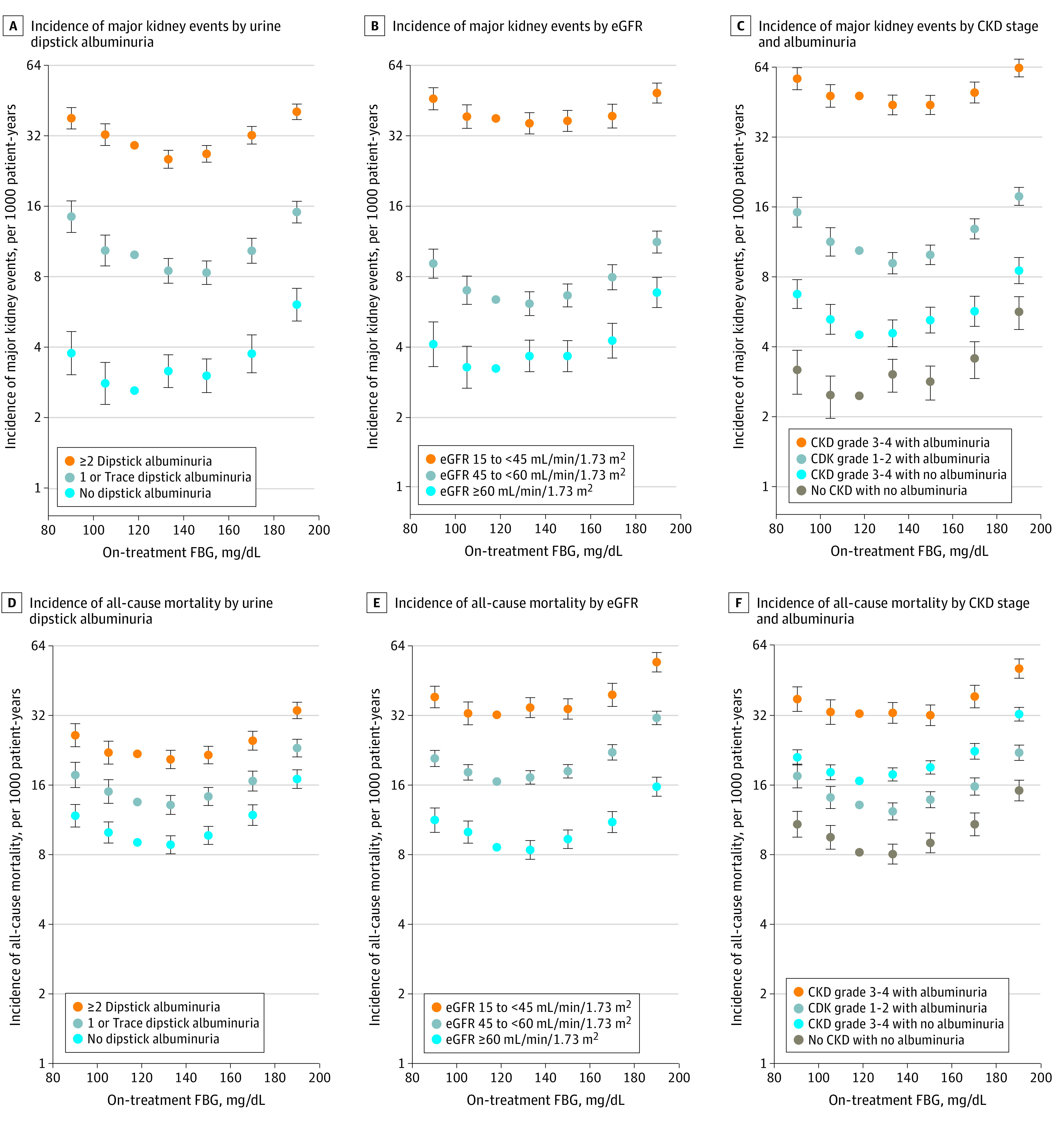
Adjusted Incidence of Major Kidney Events and All-Cause Mortality by Albuminuria and Estimated Glomerular Filtration Rate (eGFR) Incidence rates (log-scale y axis) and 95% CIs (error bars) in fasting blood glucose (FBG) level categories (ie, 80 mg/dL to <100 mg/dL, 100 mg/dL to <110 mg/dL, 110 mg/dL to <126 mg/dL, 126 mg/dL to <140 mg/dL, 140 mg/dL to <160 mg/dL, 160 mg/dL to <180 mg/dL, and 180 mg/dL to 900 mg/dL) were calculated by multiplying adjusted hazard ratios and 95% CIs by a constant to make the sum of the products of incidence rates and patient-years equal the total number of observed events. The rare FBG level of less than 80 mg/dL was excluded from the plots. The on-treatment FBG level of 110 mg/dL to <126 mg/dL was set as the reference. The major kidney event was a composite of doubling serum creatinine, end-stage kidney disease, or death from chronic kidney disease (CKD).

**Figure 4.**
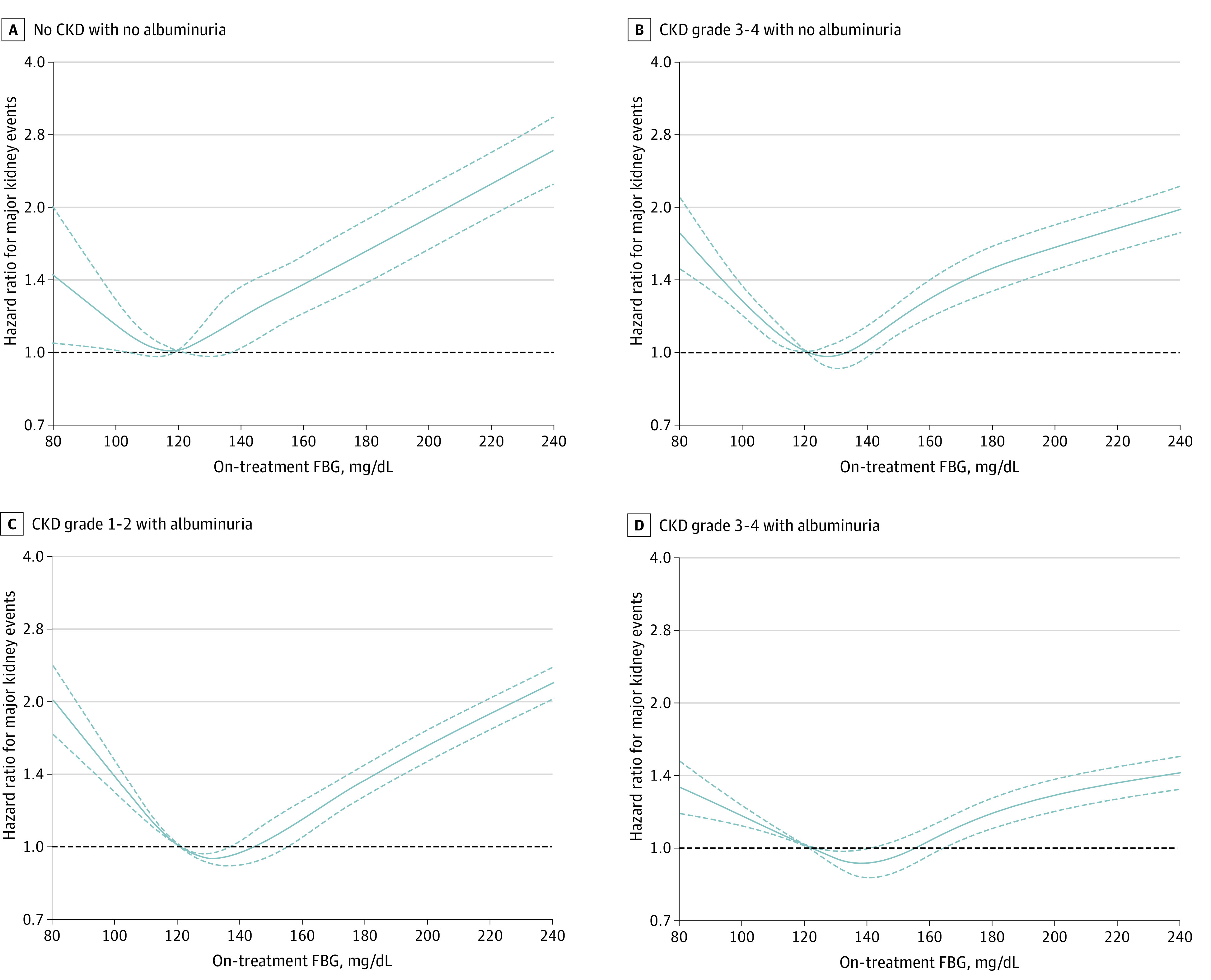
Associations of Fasting Blood Glucose (FBG) Level With Risk of Major Kidney Events by Albuminuria and Estimated Glomerular Filtration Rate (eGFR) The hazard ratios (solid lines) and 95% CIs (dotted lines) were estimated using a restricted cubic spline function. The on-treatment FBG level of 120 mg/dL was set as the reference (dashed lines). The major kidney event was a composite of doubling serum creatinine, end-stage kidney disease, or death from chronic kidney disease (CKD). Analyses were adjusted for time-varying covariates of antidiabetic use status and untreated FBG level and fixed covariates of baseline age, sex, eGFR, proteinuria, time of antidiabetic initiation, antihypertensive and statin use statuses, systolic blood pressure, body mass index (calculated as weight in kilograms divided by height in meters squared), family history of cardiovascular disease, income, smoking status, physical exercise frequency, drinking amounts, and total, high-density lipoprotein, and low-density lipoprotein cholesterol levels. CKD grades 1 to 2 and CKD grades 3 to 4 denote CKD with an eGFR of 60 mL/min/1.73 m^2^ or more and CKD with an eGFR of 15 mL/min/1.73 m^2^ to less than 60 ml/min/1.73 m^2^, respectively.

The primary outcome occurred among 13802 individuals with CKD (10.5%) and 1421 individuals with no CKD (2.8%). In subgroup analyses stratified by albuminuria or eGFR level (eTables 4 and 5 in the Supplement), the HR for major kidney events was lowest at FBG levels of 126 mg/dL to less than 160 mg/dL among participants with albuminuria and at levels of 100 mg/dL to less than 180 mg/dL among participants with an eGFR of 15 ml/min/1.73 m^2^ to less than 45 ml/min/1.73 m^2^, whereas it was lowest at 100 mg/dL to less than 126 mg/dL among participants without albuminuria or a decreased eGFR (Figure 3A and 3B; eFigure 2 in the Supplement). Among patients with an eGFR of 15 ml/min/1.73 m^2^ to less than 45 ml/min/1.73 m^2^, FBG levels of 80 mg/dL to less than 100 mg/dL (HR, 1.22; 95% CI, 1.09-1.36) and levels more than 180 mg/dL (HR, 1.29; 95% CI, 1.16-1.42) were associated with increased risk of the primary outcome compared with FBG levels of 110 mg/dL to less than 126 mg/dL. Among patients with no albuminuria, FBG levels of 80 mg/dL to less than 100 mg/dL (HR, 1.45; 95% CI, 1.17-1.79) and levels of 126 mg/dL to less than 140 mg/dL (HR, 1.21; 95% CI, 1.03-1.42) were associated with increased risk compared with FBG levels of 110 mg/dL to less than 126 mg/dL (eTable 4 in the Supplement). Among patients without decreased eGFR, FBG levels of 80 mg/dL to less than 100 mg/dL (HR, 1.27; 95% CI, 1.02-1.59) and levels of 126 mg to less than 160 mg/dL (HR, 1.13, 95% CI, 1.01-1.26) were associated with increased risk compared with FBG levels of 110 mg/dL to less than 126 mg/dL (eTable 5 in the Supplement). Among patients with no CKD, FBG levels of 80 mg/dL to less than 100 mg/dL (HR, 1.29; 95% CI, 1.01-1.65) and levels of 126 mg/dL to less than 140 mg/dL (HR, 1.23; 95% CI, 1.03-1.47) were associated with increased risk compared with FBG levels of 110 mg/dL to less than 126 mg/dL. For all-cause mortality, the risk was lowest at an FBG level of 110 mg/dL to less 140 mg/dL among most patients, while the J curve shifted to the right among participants with albuminuria but not among participants with decreased eGFR (Figure 3D and 3E; eFigure 3 in the Supplement). When analyses were performed in subgroups classified by the presence or absence of albuminuria and a decreased eGFR (eTables 6 and 7 in the Supplement), the upper threshold of the FBG level at which HRs for major kidney events increased compared with a level of 110 mg/dL to less than 126 mg/dL was increased in the order of no CKD (126 mg/dL to <140 mg/dL: HR, 1.23; 95% CI, 1.03-1.47), decreased eGFR only (140 mg/dL to <160 mg/dL: HR, 1.16; 95% CI, 1.02-1.31), albuminuria only (160 mg/dL to 180 mg/dL: HR, 1.24 ; 95% CI, 1.12-1.37), and both conditions (≥180 mg/dL: HR, 1.32; 95% CI, 1.21-1.44) (Figure 3C and Figure 4). For all-cause mortality, the upper thresholds of FBG levels were 140 mg/dL to less than 160 mg/dL among patients with no CKD (HR, 1.10; 95% CI, 1.00-1.21) and those with decreased eGFR only (HR, 1.14; 95% CI, 1.07-1.22), while the FBG thresholds were 160 mg/dL to less than 180 mg/dL among patients with albuminuria only (HR, 1.20; 95% CI, 1.00-1.45) and those with both conditions (HR, 1.19; 95% CI, 1.06-1.33) (Figure 3F; eFigure 4 in the Supplement).

### Additional Analyses

In Cox models including time-dependent interaction terms of FBG levels and a function of time, interactions were not statistically significant in most interaction terms, suggesting that there were no severe violations in the proportional hazard assumption (eTable 8 in the Supplement). When the analyses for doubling serum creatinine were performed after excluding individuals with transient creatinine doubling, the J curves were similar to those in the primary analyses (eFigure 5 in the Supplement). When the analyses for major kidney events were performed including baseline rather than time-updated FBG levels, the J curves were comparable to those of time-updated FBG levels except for the left-shifted curve among participants with a decreased eGFR (eFigure 6 in the Supplement).

## Discussion

In this nationwide cohort study of Korean adults with diabetes using antihyperglycemic agents with and without CKD with 9 years of follow-up, the on-treatment FBG level with the lowest HR for a composite of doubling serum creatinine, end-stage kidney disease, and death from CKD was 126 mg/dL to less than 160 mg/dL among patients with albuminuria and at levels of 100 mg/dL to less than 180 mg/dL among those with an eGFR of 15 ml/min/1.73 m^2^ to less than 45 ml/min/1.73 m^2^. The FBG level with the lowest HR was 100 mL/min/1.73 m^2^ to less than 126 mg/dL among patients with no CKD. The HR for new-onset albuminuria was lowest at FBG levels less than 140 mg/dL, and the risk for major cardiovascular events was lowest at 110 mg/dL to less than 140 mg/dL. All-cause mortality HRs were lowest at an FBG level of 110 mg/dL to less than 140 mg/dL among most patients, while the J curve was right-shifted among patients with albuminuria but not among patients with decreased eGFR. The key and novel finding was that among patients with diabetic kidney disease and albuminuria, an increased on-treatment FBG level was associated with favorable kidney and mortality outcomes compared with no CKD.

In clinical trials,^2,3,4^ intensive vs standard glucose control consistently decreased the incidence of diabetic kidney disease. However, the trials could not answer whether glycemic control could slow progressive kidney dysfunction in diabetic kidney disease because they excluded advanced CKD and were underpowered to assess the treatment effect among patients with baseline kidney disease. In a post hoc analysis of the Action in Diabetes and Vascular Disease: Preterax and Diamicron-MR Controlled Evaluation trial,^13^ intensive vs standard control (mean achieved FBG level, 118 mg/dL vs 141 mg/dL) was associated with decreased risk of ESKD among patients with preserved kidney function. Nonetheless, intensive glucose control did not decrease the risk of creatinine doubling in that trial,^14^ and its benefit for preventing ESKD was not found in other trials.^2,15^ There are few studies, even observational studies, that investigate the association between glycemic control and risk of kidney disease progression. In a cohort study of 23 296 patients with CKD that had nearly 4 years of follow-up, an HbA_1c_ level of less than 9% was associated with increased risk for creatinine doubling and ESKD among patients with an eGFR of 30 mL/min/1.73 m^2 ^to less than 60 mL/min/1.73 m^2^, while there was a smaller increase in risk among patients with an eGFR of 15 mL/min/1.73 m^2^ to less than 30 ml/min/1.73 m^2^.^16^ By comparison, in our longer-term and larger-scale study, the curve of on-treatment FBG level HRs for creatinine doubling and kidney failure was J shaped, and the FBG level with the nadir risk was higher among patients with albuminuria or a decreased eGFR than among patients without CKD. Furthermore, the FBG level with the lowest HR for progressive kidney dysfunction was substantially higher than that for incident albuminuria. These findings support the theory of a final common pathway to ESKD^17,18^ and may provide epidemiologic evidence that the optimal on-treatment glucose level associated with slowing the progression of diabetic kidney disease is higher than the level associated with preventing the development of diabetic kidney disease.

In this study, cardiovascular and mortality HRs were lowest at an FBG level of 110 mg/dL to less than 140 mg/dL among most patients, while the FBG level with the nadir HR for all-cause mortality was higher among patients with albuminuria but not among patients with decreased eGFR. In a post hoc analysis of the Action to Control Cardiovascular Risk in Diabetes trial,^19^ intensive vs standard glucose control (median achieved FBG level, 106 mg/dL vs 147 mg/dL) was associated with increased risk for mortality among 3636 patients with CKD but not among 6506 patients with no CKD. The intensive control–associated increase in mortality risk among individuals with CKD may be associated with 3160 patients with a urine albumin to creatinine ratio of 30 mg/g or more rather than 821 patients with an eGFR of 45 mL/min/1.73 m^2^ to less than 60 mL/min/1.73 m^2^, as suggested by our study’s funding. Thus, apart from blood glucose control, intensive blood pressure lowering and active use of renin-angiotensin system blockers and sodium-glucose cotransporter 2 inhibitors should be considered in treatment of patients with albuminuria.^20,21,22,23^ However, among patients with diabetes and CKD but no albuminuria, deliberate glycemic control may still be important to decrease overall health risks.

This study had several strengths. First, long-term outcome risks could be successfully assessed according to narrow-width glycemic levels and baseline kidney disease status in large-scale cohorts. Additionally, the study included albuminuria and decreased eGFR as representative of diabetic kidney disease. Furthermore, a timely mean of FBG levels was used as the exposure variable to assess the outcome associated with glycemic control during the study follow-up. In particular, on-treatment and untreated FBG levels were separately included in the analysis. In general, untreated FBG level shows linear associations with outcome risks,^8,24^ whereas on-treatment glucose has J-shaped risk curves.^9,10^ The inclusion of untreated blood glucose could be associated with overestimation of the benefits of glycemic control. The separated on-treatment glucose levels may better reflect treatment outcomes than unseparated levels.

### Limitations

This study also has several limitations. Given the lack of data on HbA_1c_, FBG level was used only to assess glycemic status. Although HbA_1c_ is widely used as a target of glycemic control, it cannot reflect glucose variability or extent of hypoglycemia.^25^ There is a need to consider FBG level in practice, and the information on optimal on-treatment FBG levels may be relevant in treatment of patients with CKD, among whom hypoglycemia is common.^26,27^ In addition, the biennial measurement of urine albumin and serum creatinine could potentially lead to errors in time to event analyses. However, in this 9-year follow-up study, the 1 year or 2 years earlier or later detection of albuminuria or creatinine doubling may not considerably influence the results. In addition, this study did not distinguish between type 1 and type 2 diabetes given that there were no available data to distinguish between the 2 conditions, the risk estimates could potentially be influenced by reverse causality or residual confounding, and drug-specific outcomes associated with antidiabetic treatment were not analyzed, given that many hypoglycemic drugs were used for various periods. Additionally, caution is required when applying the findings to other populations given that this study included residents in Korea aged 40 to 74 years who had no known cardiovascular or cancer disease.

## Conclusions

This study’s findings suggest that timely blood glucose control is important for preventing diabetic kidney disease and that intensive vs standard glycemic control may not be associated with greater protection for the progression of established diabetic kidney disease. An individualized and comprehensive approach is necessary for treating patients with diabetes and CKD. Nonetheless, careful glycemic control may still be associated with decreasing overall health risks among patients with CKD, particularly those with no albuminuria.

## Notes

Supplement. **eAppendix.** Covariates **eTable 1.** National Health Insurance Service Codes for Patient Treatment and Diagnosis **eTable 2.** Time-lagged Covariates for Subsequent Years **eTable 3.** Hazard Ratios of On-Treatment Fasting Blood Glucose Level in Albuminuria, Decreased Estimated Glomerular Filtration Rate, and General Populations **eTable 4.** Hazard Ratios of On-Treatment Fasting Blood Glucose Level by Albuminuria Level **eTable 5.** Hazard Ratios of On-Treatment Fasting Blood Glucose Level by Estimated Glomerular Filtration Rate **eTable 6.** Hazard Ratios of On-Treatment Fasting Blood Glucose Level Among Participants with Chronic Kidney Disease **eTable 7.** Hazard Ratios of On-Treatment Fasting Blood Glucose Level Among Participants With No Chronic Kidney Disease **eTable 8.** Time-Dependent Interactions of On-Treatment Fasting Blood Glucose Level and a Log Function of Follow-Up Time **eFigure 1.** Distribution of Follow-Up Fasting Blood Glucose Level in Albuminuria, Decreased Estimated Glomerular Filtration Rate, and General Populations **eFigure 2.** Association of On-Treatment Fasting Blood Glucose Level and Risk of Major Kidney Events by Albuminuria or Estimated Glomerular Filtration Rate Level **eFigure 3.** Association of On-Treatment Fasting Blood Glucose Level and All-Cause Mortality by Albuminuria or Estimated Glomerular Filtration Rate Level **eFigure 4.** Association of On-Treatment Fasting Blood Glucose Level and All-Cause Mortality by Presence or Absence of Albuminuria and Decreased Estimated Glomerular Filtration Rate **eFigure 5.** Association of On-Treatment Fasting Blood Glucose Level and Risk of Doubling Serum Creatinine Before and After Exclusion of Transient Creatinine Doubling **eFigure 6.** Comparison of Kidney Risks of Time-Updated and Baseline Fasting Blood Glucose Level
eReferences.
