# Supplementary material for: Evaluation of Serum Glucose and Kidney Disease Progression Among Patients With Diabetes
Source: JAMA Netw Open. 2021 Sep 29;4(9):e2127387. doi: 10.1001/jamanetworkopen.2021.27387 (PMC8482057; doi:10.1001/jamanetworkopen.2021.27387)
Supplement: Supplement. — eAppendix. Covariates eTable 1. National Health Insurance Service Codes for Patient Treatment and Diagnosis eTable 2. Time-lagged Covariates for Subsequent Years eTable 3. Hazard Ratios of On-Treatment Fasting Blood Glucose Level in Albuminuria, Decreased Estimated Glomerular Filtration Rate, and General Populations eTable 4. Hazard Ratios of On-Treatment Fasting Blood Glucose Level by Albuminuria Level eTable 5. Hazard Ratios of On-Treatment Fasting Blood Glucose Level by Estimated Glomerular Filtration Rate eTable 6. Hazard Ratios of On-Treatment Fasting Blood Glucose Level Among Participants with Chronic Kidney Disease eTable 7. Hazard Ratios of On-Treatment Fasting Blood Glucose Level Among Participants With No Chronic Kidney Disease eTable 8. Time-Dependent Interactions of On-Treatment Fasting Blood Glucose Level and a Log Function of Follow-Up Time eFigure 1. Distribution of Follow-Up Fasting Blood Glucose Level in Albuminuria, Decreased Estimated Glomerular Filtration Rate, and General Populations eFigure 2. Association of On-Treatment Fasting Blood Glucose Level and Risk of Major Kidney Events by Albuminuria or Estimated Glomerular Filtration Rate Level eFigure 3. Association of On-Treatment Fasting Blood Glucose Level and All-Cause Mortality by Albuminuria or Estimated Glomerular Filtration Rate Level eFigure 4. Association of On-Treatment Fasting Blood Glucose Level and All-Cause Mortality by Presence or Absence of Albuminuria and Decreased Estimated Glomerular Filtration Rate eFigure 5. Association of On-Treatment Fasting Blood Glucose Level and Risk of Doubling Serum Creatinine Before and After Exclusion of Transient Creatinine Doubling eFigure 6. Comparison of Kidney Risks of Time-Updated and Baseline Fasting Blood Glucose Level eReferences. [file jamanetwopen-e2127387-s001.pdf]

Supplemental Online Content

Jung HH. Evaluation of serum glucose and kidney disease progression among patients with diabetes. *JAMA Netw Open*. 2021;4(9):e2127387.
doi:10.1001/jamanetworkopen.2021.27387

eAppendix. Covariates
eTable 1. National Health Insurance Service Codes for Patient Treatment and Diagnosis
eTable 2. Time-lagged Covariates for Subsequent Years
eTable 3. Hazard Ratios of On-Treatment Fasting Blood Glucose Level in Albuminuria, Decreased Estimated Glomerular Filtration Rate, and General Populations
eTable 4. Hazard Ratios of On-Treatment Fasting Blood Glucose Level by Albuminuria Level
eTable 5. Hazard Ratios of On-Treatment Fasting Blood Glucose Level by Estimated Glomerular Filtration Rate
eTable 6. Hazard Ratios of On-Treatment Fasting Blood Glucose Level Among Participants with Chronic Kidney Disease
eTable 7. Hazard Ratios of On-Treatment Fasting Blood Glucose Level Among Participants With No Chronic Kidney Disease
eTable 8. Time-Dependent Interactions of On-Treatment Fasting Blood Glucose Level and a Log Function of Follow-Up Time
eFigure 1. Distribution of Follow-Up Fasting Blood Glucose Level in Albuminuria, Decreased Estimated Glomerular Filtration Rate, and General Populations
eFigure 2. Association of On-Treatment Fasting Blood Glucose Level and Risk of Major Kidney Events by Albuminuria or Estimated Glomerular Filtration Rate Level
eFigure 3. Association of On-Treatment Fasting Blood Glucose Level and All-Cause Mortality by Albuminuria or Estimated Glomerular Filtration Rate Level
eFigure 4. Association of On-Treatment Fasting Blood Glucose Level and All-Cause Mortality by Presence or Absence of Albuminuria and Decreased Estimated Glomerular Filtration Rate
eFigure 5. Association of On-Treatment Fasting Blood Glucose Level and Risk of Doubling Serum Creatinine Before and After Exclusion of Transient Creatinine Doubling
eFigure 6. Comparison of Kidney Risks of Time-Updated and Baseline Fasting Blood Glucose Level
eReferences.

This supplemental material has been provided by the authors to give readers additional information about their work.

eAppendix. Covariates
Baseline data were obtained from the records of nationwide health screenings performed between 2005 and 2010 generally at 2-year intervals. The covariates were determined using the averaged values for 2005–2010, except for the values of HDL and LDL cholesterols and eGFR, which were averaged for 2009–2010.

First, outlier data were excluded from the health screening records.
(a) serum creatinine <0.3 mg/dl or >15.0 mg/dl
(b) systolic blood pressure <90 mm Hg or >200 mm Hg
(c) fasting blood glucose <30 mg/dl or >900 mg/dl
(d) total cholesterol <130 mg/dl or >320 mg/dl
(e) HDL cholesterol <20 mg/dl or >100 mg/dl
(f) LDL cholesterol <30 mg/dl or >300 mg/dl
(g) body mass index <10.0 kg/m^2 or >50.0 kg/m^2
(h) alcohol drinks per drinking day >35 drinks

Baseline age was classified into 5-year age categories (40–44, 45–49, 50–54, 55–59, 60–64, 65–69, and 70–74 years). Sex, albuminuria, and family history of cardiovascular disease were categorized dichotomously.

Predicted 10-year risk of cardiovascular disease was calculated using the 2018 revised Pooled Cohort Equations[1] to compare the overall burden of conventional risk factors between groups.

Medication statuses of antihypertensives and statins were categorized into 3 groups (never, irregular, or regular). Regular use was considered when the days of prescription were for >1/2 of medication period (from the initiation of medication to the baseline), irregular use when those were for ≤1/2 of the period, and never use when those were never for ≥90 days per year.

Systolic blood pressure was categorized into 8 groups (90–105, 105–114, 115–124, 125–134, 135–144, 145–154, 155–164, or 165–200 mm Hg) primarily with 10 mm Hg intervals.

Total cholesterol was categorized into 5 groups (130–159, 160–199, 200–239, 240–279, or 280–320 mg/dl), HDL cholesterol into 3 groups (20–39, 40–59, or 60–100 mg/dl), and LDL cholesterol into 5 groups (30–99, 100–129, 130–159, 160–189, or 190–250 mg/dl), based on the ATP III classifications.[2]

Body mass index was categorized into 5 groups (underweight, 10.0–18.4; low-normal weight, 18.5–22.9; high-normal weight, 23.0–24.9, overweight, 25.0–29.9; and obesity, 30.0–50.0 kg/m^2) referring to the WHO classifications.[3]

Income levels were determined by income based insurance contributions. The vigintile values of insurance contributions were averaged from 2005 to 2010. The averaged values were categorized into 5 groups (1–4, 5–8, 9–12, 13–16, or 17–20).

Smoking status was categorized into 3 groups (never, former, or current smoker). Exercise frequency was divided into 4 categories (<1, 1–2, 3–4, or ≥5 days per week). Drinking amount was categorized into 6 groups (0.0, 0.1–3.4, 3.5–7.4, 7.5–14.4, 14.5–28.4, or ≥28.5 drinks per week) with log-scale intervals incorporating the cutoff values for the NIAAA's definition of low-risk drinking.[4]

© 2021 Jung HH et al. *JAMA Network Open.*

eTable 1. National Health Insurance Service Codes for Patient Treatment and Diagnosis

	NHIS Code
**Antidiabetic[a]**	DPP-4 inhibitor, 5008, 5011, 5023, 5029, 5070, 5071, 5137, 5185, 5186, 5196, 5205, 5206, 5207, 5238, 5247, 6133, 6164, 6191, 6242, 6273, 6303, 6304, 6305, 6306, 6320, 6356, 6357, 6396, 6418, 6419, 6420, 6450, 6453, 6484, 6485, 6486, 6499, 6500, 6501, 6541, 6646, 6647, 6648;<br>Glitazone, 3480, 4319, 4527, 4529, 4612, 4691, 4718, 4888, 4889, 4890, 4981, 5255, 5256, 5259, 6303, 6304, 6305, 6306, 6538, 6539, 6540, 6541, 6557;<br>GLP-1 agonist, 5121, 6266, 6397, 6398, 6414, 6445, 6667, 6670<br>Insulin, 1183, 1701, 1702, 1703, 1704, 1705, 1706, 1752, 1753, 2156, 2157, 3278, 4413, 4618, 4849, 4887, 5074, 6267, 6268, 6667, 6670;<br>Meglitinide, 3795, 4302, 4861, 5188, 6319, 6321, 6372, 6449;<br>Metformin, 1915, 4211, 4434, 4435, 4527, 4529, 4612, 4691, 4718, 4719, 4742, 4743, 4972, 4981, 4986, 5023, 5029, 5070, 5071, 5137, 5185, 5186, 5188, 5196, 5205, 5206, 5207, 5236, 5237, 5238, 5247, 6319, 6320, 6321, 6356, 6357, 6372, 6398, 6414, 6418, 6419, 6420, 6449, 6450, 6484, 6485, 6486, 6490, 6491, 6492, 6493, 6494, 6495, 6499, 6500, 6501, 6538, 6539, 6540, 6541, 6557, 6718, 6719, 6720, 6721, 6725, 6726, 6727, 6728, 6729, 6730, 6738, 6833, 6834;<br>SGLT2 inhibitor, 5273, 6282, 6361, 6398, 6414, 6490, 6491, 6492, 6493, 6494, 6495, 6743;<br>Sulfonylurea, 1320, 1654, 1655, 1656, 1657, 1658, 1659, 4174, 4211, 4434, 4435, 4719, 4742, 4743, 4888, 4889, 4890, 4972, 4986, 5255, 5256;<br>α-glucosidase inhibitor, 1006, 2490, 4062, 5236, 5237
**Antihypertensive[a]**	ACE inhibitor, 1042, 1147, 1229, 1330, 1409, 1516, 1635, 1734, 1845, 1968, 2113, 2219, 2224, 2310, 2350, 2351, 2424, 2622, 2623, 3787, 3859, 4028, 4403, 4407, 4408, 4470, 4471, 4472, 4486, 4487, 4536, 4537, 4660, 4901, 4979, 4992, 4993, 5016, 5104, 5562;<br>AG II antagonist, 1226, 1773, 1857, 2471, 2625, 3564, 3788, 3789, 3857, 3858, 4237, 4292, 4426, 4432, 4433, 4605, 4685, 4774, 4869, 4891, 4928, 4929, 4958, 5005, 5006, 5026, 5027, 5030, 5115, 5116, 5117, 5136, 5139, 5152, 5197, 5198, 5199, 5200, 5201, 5209, 5212, 5213, 5214, 5220, 5222, 5223, 5224, 5226, 5227, 5228, 5229, 5230, 5231, 5232, 5233, 5234, 5240, 5241, 5250, 5251, 5252, 5253, 5263, 5264, 5265, 5268, 5269, 5270, 5271, 5475, 5476, 5477, 5478, 5479, 5480, 5822, 5824, 6231, 6294, 6295, 6296, 6297, 6298, 6299, 6300, 6301, 6302, 6313, 6316, 6317, 6328, 6329, 6330, 6349, 6350, 6351, 6352, 6374, 6375, 6376, 6441, 6442, 6448, 6519, 6520, 6521, 6527, 6529, 6530, 6531, 6624, 6628, 6629, 6630, 6635, 6636, 6637, 6638, 6735, 6736, 6827, 6828, 6829;<br>Ca-antagonist, 1076, 1140, 1151, 1331, 1457, 1575, 1789, 1803, 1820, 1880, 2010, 2011, 2017, 2024, 2476, 2624, 3562, 4412, 4470, 4471, 4472, 4598, 4599, 4646, 4660, 4708, 4723, 4724, 4725, 4762, 4797, 4832, 4865, 4928, 4929, 4958, 4959, 5005, 5006, 5027, 5030, 5115, 5116, 5117, 5139, 5189, 5197, 5198, 5199, 5200, 5201, 5212, 5213, 5214, 5222, 5223, 5224, 5226, 5227, 5228, 5229, 5230, 5231, 5232, 5233, 5234, 5282, 5475, 5476, 5477, 5478, 5479, 5480, 5822, 5824, 6145, 6231, 6294, 6295, 6296, 6313, 6328, 6329, 6330, 6374, 6375, 6376, 6448, 6519, 6520, 6521, 6527, 6529, 6530, 6531, 6628, 6629, 6630, 6635, 6636, 6637, 6638, 6827, 6828, 6829;<br>Central sympatholytic, 1365, 1927, 1975, 2231, 2631;<br>Direct vasodilator, 1707, 1961, 2293, 4236, 4606;<br>Thiazide, 1708, 1744, 2447, 2620, 2621, 2622, 2623, 2625, 2626, 2627, 2628, 2630, 3564, 3787, 3789, 3857, 3858, 4237, 4273, 4274, 4403, 4407, 4408, 4426, 4432, 4433, 4486, 4487, 4513, 4536, 4537, 4554, 4555, 4605, 4698, 4699, 4700, 4774, 4869, 4891, 4901, 4979, 4992, 4993, 5026, 5136, 5197, 5198, 5200, 5220, 5268, 5562, 6628, 6629, 6630, 6635, 6636, 6637, 6638, 6735, 6736, 6827, 6828, 6829;<br>α antagonist, 1200, 1491, 1749, 2168, 2355, 2629, 4414, 4834;<br>β blocker, 1079, 1114, 1168, 1170, 1179, 1250, 1370, 1802, 2023, 2098, 2620, 2621, 2624, 2626, 4273, 4274, 4554, 4555, 4601, 4602, 4698, 4699, 4700, 4831, 4895
**Statin[a]**	Atorvastatin, 1115, 4723, 4724, 4725, 5022, 5189, 5240, 5241, 5270, 5271, 6145, 6338, 6339, 6346, 6348, 6718, 6719, 6720, 6721, 6738;<br>Cerivastatin, 1304;<br>Fluvastatin, 1624;<br>Lovastatin, 1858, 1859;<br>Pitavastatin, 4709, 6349, 6350, 6351, 6352, 6793;<br>Pravastatin, 2166, 5193;<br>Rosuvastatin, 4540, 5250, 5251, 5252, 5253, 5263, 5264, 5265, 5269, 6297, 6298, 6299, 6300, 6301, 6302, 6316, 6317, 6407, 6408, 6409, 6441, 6442, 6634, 6646, 6647, 6648, 6725, 6726, 6727, 6728, 6729, 6730, 6833, 6834;<br>Simvastatin, 2278, 4710, 4711, 5078, 6314, 6315
**Renal replacement therapy**	Haemodialysis, O7020, O9991;<br>Peritoneal dialysate, 3214, 3216, 3218, 3220, 3221, 3222, 3223, 3224, 3225, 3226, 3240, 3243, 3244, 3245, 3248, 3249, 3250, 3251, 3252, 3253, 3254, 3255, 3256, 3257, 3259, 3260, 3496, 3497, 3498, 3499, 3500, 3501, 3502, 3503, 3506, 3507, 3508, 3509, 3510, 3511, 3512, 3513, 3514, 3515, 3516, 3517, 3518, 3519, 3520, 3521, 3522, 3523, 3524, 3525, 3526, 3527, 3528, 3529, 3530, 3531, 3532, 3533, 3534, 3535, 3536, 3537, 3538, 3539, 3540, 3541, 3601, 3602, 3603, 3659, 3660, 3661, 3662, 3663, 3666, 3667, 3668, 3669, 3808, 3809, 4008, 4009, 4010, 4095, 4096, 4097, 4098, 4099, 4229, 4230, 4231, 4232, 4234, 4235, 4311, 4312, 4313, 4490, 4491, 4492, 4498, 4499, 4500, 4637, 4639, 4640, 4647, 4648, 4845, 4846, 4847, 4935, 4936, 4949, 4950, 4951, 5096, 5097, 5098, 5107, 5108, 5109, 5110, 5111, 5135, 5161, 5162, 5163, 6657, 6658, 6659, 6816, 6817, 6818, 6819, 6820, 6821;<br>Kidney transplantation, R3280
**CCU admission**	NHIS clause code, 02; and NHIS item code, 03
**Coronary revascularization**	Percutaneous coronary angioplasty, M6551, M6552, M6553, M6554;<br>Percutaneous coronary stent insertion, M6561, M6562, M6563, M6564, M6565, M6566, M6567;<br>Percutaneous coronary thrombolysis or thrombectomy, M6634, M6638;<br>Percutaneous coronary atherectomy, M6571, M6572;<br>Coronary artery bypass graft, O1647, OA641, OA642;<br>Coronary artery endarterectomy, O1830
**Cerebral or carotid revascularizatio**	Percutaneous cerebral angioplasty, M6593, M6599;<br>Percutaneous cerebral stent insertion, M6601;<br>Percutaneous cerebral thrombolysis or thrombectomy, M6630, M6636;

© 2021 Jung HH et al. *JAMA Network Open.*

**n**	Percutaneous carotid angioplasty, M6594;<br>Percutaneous carotid stent insertion, M6602;<br>Percutaneous carotid thrombolysis or thrombectomy, M6635, M6637;<br>Transluminal carotid atherectomy, O0226;<br>Carotid endarterectomy, O0227
**Diagnosis**	Acute ischemic stroke, I63–I66;<br>Acute coronary syndrome, I21–I24;<br>Cardiovascular disease, I00–I99;<br>Chronic kidney disease, N18;<br>Kidney transplant status, Z94.0

[a] The codes for combination drugs are repeated in corresponding cells.
ACE, angiotensin-converting-enzyme; AG, angiotensin; CCU, critical care unit; DPP-4 dipeptidyl peptidase-4; GLP-1, glucagon-like peptide-1; NHIS, National Health Insurance Service; SGLT2, sodium-glucose transport protein 2.

© 2021 Jung HH et al. *JAMA Network Open.*

eTable 2. Time-lagged Covariates for Subsequent Years

**Year of Outcome Variable**	**Year of Time-Varying Covariate**		**Period for Fixed Covariate**
**Clinical Endpoint**	**Time-Averaged FBG**	**Antidiabetic Use Status**	**Baseline Value**
2011	2005–2010	2010	2005–2010
2012	2005–2011	2011	2005–2010
2013	2005–2012	2012	2005–2010
2014	2005–2013	2013	2005–2010
2015	2005–2014	2014	2005–2010
2016	2005–2015	2015	2005–2010
2017	2005–2016	2016	2005–2010
2018	2005–2017	2017	2005–2010
2019	2005–2018	2018	2005–2010

FBG, fasting blood glucose.

© 2021 Jung HH et al. *JAMA Network Open.*

eTable 3. Hazard Ratios of On-Treatment Fasting Blood Glucose Level in Albuminuria, Decreased Estimated Glomerular Filtration Rate, and General Populations[a]

Outcome	CKD with Dipstick Albuminuria		CKD with eGFR <60 ml/min/1.73 m^2		General Population	
FBG	no. of event / patient-yr	hazard ratio (95% CI)	no. of event / patient-yr	hazard ratio (95% CI)	no. of event / patient-yr	hazard ratio (95% CI)
**Major kidney events[b]**						
<80 mg/dl	145 / 2776	1.92 (1.62–2.28)	148 / 5335	1.61 (1.36–1.91)	26 / 2893	1.58 (0.86–2.90)
80–<100 mg/dl	743 / 25972	1.40 (1.29–1.53)	814 / 53661	1.30 (1.20–1.42)	195 / 35462	1.18 (0.93–1.50)
100–<110 mg/dl	728 / 36427	1.09 (1.00–1.19)	727 / 65301	1.06 (0.97–1.16)	198 / 49892	1.08 (0.86–1.35)
110–<126 mg/dl	1624 / 94672	1.00	1424 / 139532	1.00	448 / 120943	1.00
126–<140 mg/dl	1416 / 95870	0.87 (0.81–0.94)	1125 / 107785	0.96 (0.88–1.03)	435 / 105114	0.84 (0.69–1.02)
140–<160 mg/dl	1803 / 117326	0.90 (0.84–0.96)	1265 / 102855	1.01 (0.93–1.08)	459 / 106019	0.90 (0.74–1.09)
160–<180 mg/dl	1525 / 80701	1.10 (1.03–1.18)	869 / 55867	1.13 (1.04–1.23)	330 / 59492	1.28 (1.04–1.57)
≥180 mg/dl	3136 / 119779	1.49 (1.40–1.58)	1676 / 68642	1.50 (1.39–1.61)	696 / 72532	1.61 (1.33–1.95)
**Doubling serum creatinine**						
<80 mg/dl	91 / 2742	1.60 (1.29–1.98)	78 / 5113	1.15 (0.91–1.45)	15 / 2724	1.53 (0.91–2.57)
80–<100 mg/dl	498 / 23390	1.37 (1.24–1.53)	469 / 46344	1.23 (1.10–1.38)	157 / 31435	1.45 (1.20–1.75)
100–<110 mg/dl	500 / 31779	1.09 (0.98–1.21)	435 / 55027	1.06 (0.94–1.19)	162 / 43622	1.06 (0.89–1.28)
110–<126 mg/dl	1170 / 82600	1.00	872 / 116910	1.00	375 / 105354	1.00
126–<140 mg/dl	1040 / 82824	0.88 (0.80–0.95)	701 / 89797	0.96 (0.87–1.06)	376 / 90835	1.09 (0.95–1.26)
140–<160 mg/dl	1393 / 101188	0.93 (0.86–1.01)	791 / 85663	1.00 (0.91–1.10)	395 / 91578	1.08 (0.93–1.24)
160–<180 mg/dl	1177 / 70003	1.12 (1.03–1.22)	539 / 46539	1.10 (0.99–1.23)	286 / 51214	1.27 (1.09–1.49)
≥180 mg/dl	2266 / 105499	1.44 (1.34–1.55)	1010 / 58693	1.40 (1.28–1.54)	555 / 62821	1.94 (1.69–2.22)
**Kidney failure[c]**						
<80 mg/dl	102 / 2847	1.49 (1.21–1.82)	122 / 5384	1.52 (1.26–1.83)	12 / 2901	2.06 (1.14–3.72)
80–<100 mg/dl	563 / 26631	1.31 (1.18–1.45)	687 / 54193	1.30 (1.18–1.43)	84 / 35860	1.34 (1.03–1.75)
100–<110 mg/dl	549 / 37430	1.07 (0.96–1.18)	610 / 65954	1.06 (0.96–1.17)	83 / 50420	1.04 (0.80–1.35)
110–<126 mg/dl	1168 / 97041	1.00	1181 / 140918	1.00	176 / 122273	1.00
126–<140 mg/dl	957 / 98133	0.87 (0.80–0.94)	904 / 108907	0.94 (0.86–1.02)	143 / 106399	0.89 (0.71–1.11)
140–<160 mg/dl	1148 / 120239	0.86 (0.80–0.94)	1009 / 103940	0.98 (0.90–1.06)	151 / 107421	0.79 (0.63–0.98)
160–<180 mg/dl	966 / 82882	1.07 (0.98–1.16)	714 / 56582	1.12 (1.02–1.23)	133 / 60341	1.08 (0.86–1.36)
≥180 mg/dl	2001 / 123276	1.45 (1.34–1.56)	1358 / 69495	1.46 (1.34–1.58)	346 / 73678	2.08 (1.71–2.51)
**Major cardiovascular events**						
<80 mg/dl	53 / 3105	1.79 (1.35–2.37)	74 / 5650	1.51 (1.19–1.92)	14 / 2912	1.09 (0.64–1.85)
80–<100 mg/dl	269 / 27976	1.14 (0.99–1.32)	512 / 55420	1.18 (1.07–1.32)	182 / 35791	1.22 (1.03–1.45)
100–<110 mg/dl	318 / 38544	1.04 (0.92–1.19)	546 / 66785	1.08 (0.98–1.20)	239 / 50206	1.17 (1.00–1.37)
110–<126 mg/dl	727 / 98902	1.00	1056 / 142205	1.00	478 / 121588	1.00
126–<140 mg/dl	747 / 99343	1.06 (0.96–1.17)	878 / 109972	1.07 (0.98–1.17)	423 / 105757	1.03 (0.90–1.17)
140–<160 mg/dl	983 / 121678	1.17 (1.06–1.29)	986 / 105176	1.24 (1.13–1.35)	505 / 106735	1.22 (1.07–1.38)
160–<180 mg/dl	707 / 84323	1.24 (1.12–1.38)	605 / 57699	1.37 (1.24–1.51)	352 / 59959	1.53 (1.33–1.76)
≥180 mg/dl	1337 / 126354	1.61 (1.47–1.77)	999 / 71998	1.78 (1.63–1.95)	505 / 73589	1.87 (1.64–2.13)
**All-cause mortality**						
<80 mg/dl	123 / 3162	1.63 (1.36–1.96)	218 / 5732	1.73 (1.51–1.99)	79 / 2933	2.34 (1.86–2.94)
80–<100 mg/dl	776 / 28391	1.27 (1.16–1.38)	1443 / 56213	1.24 (1.16–1.32)	514 / 36099	1.30 (1.17–1.44)
100–<110 mg/dl	840 / 39073	1.06 (0.98–1.15)	1450 / 67673	1.07 (1.01–1.14)	612 / 50613	1.13 (1.02–1.24)
110–<126 mg/dl	1881 / 100391	1.00	2838 / 144197	1.00	1259 / 122688	1.00
126–<140 mg/dl	1718 / 100886	0.96 (0.90–1.03)	2301 / 111596	1.05 (1.00–1.11)	1035 / 106771	0.97 (0.89–1.05)
140–<160	2131 / 123670	1.03 (0.96–1.09)	2283 / 106951	1.10 (1.04–1.16)	1135 / 107846	1.07 (0.99–1.16)

© 2021 Jung HH et al. *JAMA Network Open.*

mg/dl						
160–<180 mg/dl	1597 / 85633	1.20 (1.12–1.28)	1464 / 58774	1.31 (1.22–1.39)	728 / 60686	1.28 (1.16–1.40)
≥180 mg/dl	3061 / 128708	1.65 (1.55–1.75)	2502 / 73543	1.82 (1.72–1.92)	1163 / 74550	1.81 (1.67–1.97)
	**eGFR ≥60 ml/min/1.73 m^2**		**eGFR <60 ml/min/1.73 m^2**		**General Population**	
**New-onset albuminuria**						
<80 mg/dl	46 / 1627	2.13 (1.59–2.87)	123 / 2660	1.58 (1.32–1.90)	57 / 1881	1.97 (1.51–2.57)
80–<100 mg/dl	312 / 22288	0.97 (0.86–1.10)	933 / 31555	1.11 (1.03–1.20)	423 / 25700	1.06 (0.95–1.18)
100–<110 mg/dl	483 / 32539	1.04 (0.94–1.16)	1090 / 39611	1.04 (0.96–1.11)	607 / 36604	1.04 (0.95–1.15)
110–<126 mg/dl	1199 / 79081	1.00	2282 / 83922	1.00	1433 / 87792	1.00
126–<140 mg/dl	1173 / 68029	1.02 (0.95–1.11)	1797 / 62089	1.05 (0.98–1.11)	1355 / 74110	1.10 (1.02–1.18)
140–<160 mg/dl	1285 / 66208	1.13 (1.04–1.22)	1817 / 55690	1.15 (1.08–1.23)	1470 / 72006	1.19 (1.10–1.28)
160–<180 mg/dl	830 / 35162	1.37 (1.25–1.49)	1027 / 28122	1.28 (1.18–1.37)	921 / 37993	1.42 (1.30–1.54)
≥180 mg/dl	1335 / 39526	2.08 (1.92–2.25)	1495 / 30101	1.80 (1.69–1.93)	1495 / 42518	2.10 (1.95–2.26)

[a] The hazard ratios were adjusted for time-varying covariates of antidiabetic use status and untreated FBG and baseline covariates of age, sex, eGFR, proteinuria, time of antihypertensive initiation, antihypertensive and statin use statuses, fasting glucose, total cholesterol, HDL cholesterol, LDL cholesterol, body mass index, family history of cardiovascular disease, income, smoking, physical exercise, and drinking.
[b] Major kidney event was a composite of kidney failure or doubling of serum creatinine.
[c] Kidney failure was identified as dialysis for ≥90 days per year, kidney transplantation, or death from CKD.
CKD, chronic kidney disease; eGFR, estimated glomerular filtration rate; FBG, fasting blood glucose..

© 2021 Jung HH et al. *JAMA Network Open.*

eTable 4. Hazard Ratios of On-Treatment Fasting Blood Glucose Level by Albuminuria Level[a]

Outcome	Dipstick Albumin ≥2+		Dipstick Albumin 1+ or trace		No Albuminuria	
FBG	no. of event / patient-yr	hazard ratio (95% CI)	no. of event / patient-yr	hazard ratio (95% CI)	no. of event / patient-yr	hazard ratio (95% CI)
**Major kidney events[b]**						
<80 mg/dl	105 / 1337	1.68 (1.37–2.06)	40 / 1439	2.32 (1.68–3.20)	14 / 2657	1.88 (1.09–3.22)
80–<100 mg/dl	518 / 10839	1.31 (1.18–1.45)	225 / 15133	1.46 (1.25–1.70)	125 / 32957	1.45 (1.17–1.79)
100–<110 mg/dl	494 / 14226	1.11 (1.00–1.24)	234 / 22201	1.04 (0.90–1.22)	129 / 46231	1.07 (0.87–1.32)
110–<126 mg/dl	1056 / 36139	1.00	568 / 58533	1.00	286 / 111727	1.00
126–<140 mg/dl	931 / 37825	0.87 (0.80–0.95)	485 / 58045	0.85 (0.76–0.97)	298 / 95457	1.21 (1.03–1.42)
140–<160 mg/dl	1217 / 46672	0.92 (0.85–1.00)	586 / 70654	0.84 (0.75–0.94)	283 / 94390	1.16 (0.98–1.36)
160–<180 mg/dl	1040 / 33824	1.10 (1.01–1.20)	485 / 46877	1.04 (0.92–1.18)	191 / 51106	1.44 (1.19–1.73)
≥180 mg/dl	2152 / 54411	1.39 (1.29–1.50)	984 / 65368	1.53 (1.37–1.70)	368 / 60486	2.33 (1.99–2.74)
**Doubling serum creatinine**						
<80 mg/dl	63 / 1362	1.39 (1.07–1.80)	28 / 1380	2.10 (1.43–3.08)	11 / 2484	1.49 (0.81–2.73)
80–<100 mg/dl	350 / 9994	1.32 (1.16–1.51)	148 / 13396	1.31 (1.08–1.58)	111 / 29186	1.39 (1.11–1.74)
100–<110 mg/dl	320 / 12462	1.08 (0.95–1.24)	180 / 19317	1.09 (0.92–1.29)	113 / 40477	1.03 (0.82–1.28)
110–<126 mg/dl	727 / 31637	1.00	443 / 50963	1.00	262 / 97244	1.00
126–<140 mg/dl	666 / 32853	0.88 (0.79–0.97)	374 / 49971	0.84 (0.73–0.97)	274 / 82562	1.21 (1.02–1.43)
140–<160 mg/dl	926 / 40286	0.97 (0.88–1.07)	467 / 60902	0.84 (0.74–0.96)	255 / 81542	1.13 (0.95–1.34)
160–<180 mg/dl	787 / 29566	1.13 (1.02–1.25)	390 / 40437	1.05 (0.91–1.20)	172 / 43939	1.44 (1.18–1.75)
≥180 mg/dl	1537 / 48515	1.35 (1.23–1.48)	729 / 56984	1.44 (1.27–1.62)	309 / 52137	2.20 (1.85–2.62)
**Kidney failure[c]**						
<80 mg/dl	76 / 1376	1.31 (1.03–1.66)	26 / 1471	1.84 (1.23–2.75)	3 / 2664	2.31 (0.71–7.49)
80–<100 mg/dl	410 / 11237	1.16 (1.03–1.31)	153 / 15394	1.57 (1.29–1.91)	29 / 33299	1.93 (1.21–3.08)
100–<110 mg/dl	407 / 14815	1.08 (0.96–1.22)	142 / 22615	1.03 (0.85–1.26)	30 / 46674	1.50 (0.95–2.38)
110–<126 mg/dl	850 / 37415	1.00	318 / 59626	1.00	47 / 112798	1.00
126–<140 mg/dl	709 / 39094	0.86 (0.78–0.95)	248 / 59039	0.84 (0.71–0.99)	50 / 96544	1.25 (0.84–1.86)
140–<160 mg/dl	862 / 48363	0.86 (0.78–0.95)	286 / 71876	0.82 (0.70–0.96)	55 / 95516	1.28 (0.86–1.89)
160–<180 mg/dl	719 / 35130	1.03 (0.94–1.14)	247 / 47752	1.07 (0.91–1.27)	46 / 51725	1.90 (1.26–2.86)
≥180 mg/dl	1469 / 56485	1.30 (1.19–1.41)	532 / 66791	1.69 (1.46–1.95)	124 / 61293	4.25 (3.00–6.01)
**Major cardiovascular events**						
<80 mg/dl	27 / 1574	1.32 (0.89–1.95)	26 / 1531	2.53 (1.69–3.78)	12 / 2653	1.14 (0.64–2.03)
80–<100 mg/dl	152 / 12417	1.03 (0.86–1.25)	117 / 15559	1.24 (1.00–1.53)	153 / 33125	1.24 (1.03–1.50)
100–<110 mg/dl	164 / 15892	0.95 (0.79–1.14)	154 / 22652	1.16 (0.95–1.40)	206 / 46374	1.21 (1.03–1.44)
110–<126 mg/dl	394 / 39222	1.00	333 / 59680	1.00	402 / 111971	1.00
126–<140 mg/dl	381 / 40498	0.97 (0.84–1.11)	366 / 58845	1.15 (0.99–1.34)	359 / 95810	1.07 (0.92–1.23)
140–<160 mg/dl	510 / 50117	1.07 (0.93–1.22)	473 / 71561	1.27 (1.10–1.46)	406 / 94738	1.24 (1.08–1.43)
160–<180 mg/dl	350 / 36688	1.02 (0.88–1.18)	357 / 47635	1.50 (1.29–1.74)	267 / 51236	1.57 (1.34–1.84)
≥180 mg/dl	752 / 59399	1.39 (1.23–1.58)	585 / 66955	1.83 (1.59–2.11)	379 / 60790	2.02 (1.75–2.34)
**All-cause mortality**						
<80 mg/dl	82 / 1604	1.70 (1.35–2.13)	41 / 1558	1.44 (1.05–1.97)	72 / 2667	2.49 (1.96–3.17)
80–<100 mg/dl	433 / 12625	1.20 (1.07–1.35)	343 / 15766	1.31 (1.15–1.48)	442 / 33373	1.30 (1.17–1.46)
100–<110 mg/dl	433 / 16123	1.01 (0.90–1.14)	407 / 22950	1.11 (0.99–1.25)	513 / 46740	1.11 (1.00–1.23)
110–<126 mg/dl	967 / 40000	1.00	914 / 60391	1.00	1083 / 112882	1.00
126–<140 mg/dl	889 / 41287	0.95 (0.86–1.04)	829 / 59599	0.97 (0.88–1.07)	877 / 96657	0.98 (0.89–1.07)
140–<160 mg/dl	1095 / 51101	0.99 (0.91–1.08)	1036 / 72569	1.06 (0.97–1.16)	915 / 95632	1.07 (0.98–1.17)

© 2021 Jung HH et al. *JAMA Network Open.*

160–<180 mg/dl	856 / 37352	1.14 (1.04–1.25)	741 / 48281	1.23 (1.12–1.36)	573 / 51826	1.31 (1.19–1.46)
≥180 mg/dl	1743 / 60704	1.54 (1.42–1.67)	1318 / 68004	1.71 (1.57–1.87)	875 / 61507	1.88 (1.71–2.06)

[a] The hazard ratios were adjusted for time-varying covariates of antidiabetic use status and untreated FBG and baseline covariates of age, sex, eGFR, proteinuria, time of antihypertensive initiation, antihypertensive and statin use statuses, fasting glucose, total cholesterol, HDL cholesterol, LDL cholesterol, body mass index, family history of cardiovascular disease, income, smoking, physical exercise, and drinking.
[b] Major kidney event was a composite of kidney failure or doubling of serum creatinine.
[c] Kidney failure was identified as dialysis for ≥90 days per year, kidney transplantation, or death from chronic kidney disease.
FBG, fasting blood glucose; eGFR, estimated glomerular filtration rate.

© 2021 Jung HH et al. *JAMA Network Open.*

eTable 5. Hazard Ratios of On-Treatment Fasting Blood Glucose Level by Estimated Glomerular Filtration Rate[a]

**Outcome**	**15 to <45 ml/min/1.73 m²**		**45 to <60 ml/min/1.73 m²**		**≥60 ml/min/1.73 m²**	
FBG	no. of event / patient-yr	hazard ratio (95% CI)	no. of event / patient-yr	hazard ratio (95% CI)	no. of event / patient-yr	hazard ratio (95% CI)
**Major kidney events[b]**						
<80 mg/dl	105 / 1621	1.38 (1.13–1.70)	43 / 3714	2.08 (1.53–2.85)	14 / 2378	1.89 (1.10–3.23)
80–<100 mg/dl	540 / 11905	1.22 (1.09–1.36)	274 / 41756	1.43 (1.24–1.65)	108 / 29715	1.27 (1.02–1.59)
100–<110 mg/dl	437 / 11742	1.02 (0.91–1.14)	290 / 53559	1.10 (0.95–1.26)	126 / 43151	1.01 (0.82–1.25)
110–<126 mg/dl	821 / 23928	1.00	603 / 115604	1.00	309 / 106498	1.00
126–<140 mg/dl	608 / 18269	0.95 (0.86–1.06)	517 / 89516	0.96 (0.85–1.08)	328 / 94538	1.13 (0.97–1.32)
140–<160 mg/dl	654 / 18074	0.98 (0.88–1.08)	611 / 84781	1.04 (0.93–1.17)	344 / 95435	1.13 (0.97–1.32)
160–<180 mg/dl	425 / 10305	1.02 (0.91–1.15)	444 / 45562	1.25 (1.10–1.41)	243 / 53781	1.32 (1.11–1.56)
≥180 mg/dl	839 / 13845	1.29 (1.16–1.42)	837 / 54797	1.77 (1.59–1.97)	511 / 65874	2.12 (1.83–2.47)
**Doubling serum creatinine**						
<80 mg/dl	48 / 1686	0.87 (0.65–1.18)	30 / 3427	1.90 (1.31–2.76)	12 / 2212	1.60 (0.89–2.85)
80–<100 mg/dl	287 / 10565	1.13 (0.97–1.31)	182 / 35779	1.32 (1.11–1.57)	103 / 26457	1.27 (1.02–1.60)
100–<110 mg/dl	240 / 10162	1.03 (0.88–1.20)	195 / 44865	1.08 (0.91–1.28)	117 / 38011	1.00 (0.81–1.24)
110–<126 mg/dl	450 / 20315	1.00	422 / 96595	1.00	292 / 93214	1.00
126–<140 mg/dl	333 / 15471	0.92 (0.80–1.07)	368 / 74326	0.99 (0.86–1.14)	307 / 82064	1.13 (0.96–1.33)
140–<160 mg/dl	361 / 15576	0.91 (0.79–1.05)	430 / 70087	1.08 (0.94–1.23)	326 / 82680	1.15 (0.98–1.35)
160–<180 mg/dl	236 / 9000	0.95 (0.81–1.11)	303 / 37539	1.24 (1.06–1.44)	225 / 46454	1.33 (1.11–1.59)
≥180 mg/dl	466 / 12650	1.18 (1.03–1.35)	544 / 46043	1.63 (1.43–1.86)	435 / 57139	2.02 (1.73–2.36)
**Kidney failure[c]**						
<80 mg/dl	99 / 1647	1.40 (1.13–1.73)	23 / 3737	1.72 (1.12–2.62)	2 / 2383	1.30 (0.31–5.38)
80–<100 mg/dl	506 / 12100	1.22 (1.09–1.36)	181 / 42093	1.46 (1.22–1.74)	16 / 30040	1.02 (0.59–1.77)
100–<110 mg/dl	416 / 11977	1.03 (0.91–1.16)	194 / 53977	1.10 (0.93–1.30)	21 / 43621	0.84 (0.52–1.38)
110–<126 mg/dl	776 / 24360	1.00	405 / 116558	1.00	67 / 107656	1.00
126–<140 mg/dl	559 / 18559	0.94 (0.84–1.05)	345 / 90348	0.92 (0.80–1.07)	53 / 95676	0.73 (0.51–1.05)
140–<160 mg/dl	608 / 18346	0.98 (0.88–1.09)	401 / 85594	0.95 (0.83–1.09)	65 / 96736	0.75 (0.53–1.05)
160–<180 mg/dl	400 / 10474	1.04 (0.92–1.18)	314 / 46108	1.19 (1.02–1.38)	65 / 54553	1.07 (0.76–1.52)
≥180 mg/dl	760 / 14118	1.26 (1.14–1.40)	598 / 55368	1.73 (1.52–1.98)	188 / 66924	2.22 (1.66–2.99)
**Major cardiovascular events**						
<80 mg/dl	36 / 1898	1.58 (1.12–2.24)	38 / 3752	1.46 (1.05–2.02)	10 / 2366	1.14 (0.61–2.14)
80–<100 mg/dl	186 / 13397	1.16 (0.96–1.39)	326 / 42023	1.20 (1.05–1.37)	131 / 29848	1.23 (1.01–1.51)
100–<110 mg/dl	172 / 13132	1.11 (0.92–1.34)	374 / 53653	1.08 (0.95–1.22)	179 / 43347	1.17 (0.98–1.40)
110–<126 mg/dl	305 / 26424	1.00	751 / 115781	1.00	370 / 106828	1.00
126–<140 mg/dl	256 / 20099	1.12 (0.95–1.32)	622 / 89873	1.05 (0.94–1.17)	338 / 94942	1.02 (0.88–1.19)
140–<160 mg/dl	288 / 19987	1.27 (1.08–1.49)	698 / 85189	1.22 (1.10–1.35)	395 / 95963	1.19 (1.04–1.38)
160–<180 mg/dl	164 / 11719	1.21 (1.00–1.47)	441 / 45980	1.43 (1.27–1.61)	292 / 54078	1.59 (1.36–1.86)
≥180 mg/dl	297 / 16239	1.56 (1.32–1.84)	702 / 55759	1.88 (1.69–2.09)	408 / 66475	1.91 (1.65–2.22)
**All-cause mortality**						
<80 mg/dl	92 / 1945	1.45 (1.17–1.80)	126 / 3787	2.00 (1.67–2.40)	52 / 2385	2.17 (1.64–2.88)
80–<100 mg/dl	556 / 13678	1.19 (1.07–1.33)	887 / 42535	1.26 (1.16–1.36)	368 / 30078	1.31 (1.16–1.48)
100–<110 mg/dl	453 / 13352	1.02 (0.91–1.14)	997 / 54321	1.10 (1.02–1.18)	468 / 43664	1.16 (1.04–1.30)
110–<126 mg/dl	878 / 26856	1.00	1960 / 117341	1.00	960 / 107773	1.00
126–<140 mg/dl	705 / 20500	1.08 (0.97–1.19)	1596 / 91096	1.04 (0.98–1.12)	826 / 95779	0.98 (0.89–1.07)
140–<160 mg/dl	679 / 20428	1.06 (0.96–1.17)	1604 / 86523	1.11 (1.04–1.19)	907 / 96875	1.08 (0.99–1.19)

© 2021 Jung HH et al. *JAMA Network Open.*

160–<180 mg/dl	448 / 11959	1.22 (1.09–1.37)	1016 / 46815	1.34 (1.24–1.45)	584 / 54702	1.29 (1.16–1.43)
≥180 mg/dl	847 / 16688	1.69 (1.53–1.86)	1655 / 56846	1.88 (1.76–2.01)	926 / 67306	1.83 (1.66–2.01)

[a] The hazard ratios were adjusted for time-varying covariates of antidiabetic use status and untreated FBG and baseline covariates of age, sex, eGFR, proteinuria, time of antihypertensive initiation, antihypertensive and statin use statuses, fasting glucose, total cholesterol, HDL cholesterol, LDL cholesterol, body mass index, family history of cardiovascular disease, income, smoking, physical exercise, and drinking.
[b] Major kidney event was a composite of kidney failure or doubling of serum creatinine.
[c] Kidney failure was identified as dialysis for ≥90 days per year, kidney transplantation, or death from chronic kidney disease.
FBG, fasting blood glucose; eGFR, estimated glomerular filtration rate.

© 2021 Jung HH et al. *JAMA Network Open.*

eTable 6. Hazard Ratios of On-Treatment Fasting Blood Glucose Level Among Participants with Chronic Kidney Disease[a]

Outcome	CKD G1-2 with Albuminuria		CKD G3-4 with No Albuminuria		CKD G3-4 with Albuminuria	
FBG	no. of event / patient-yr	hazard ratio (95% CI)	no. of event / patient-yr	hazard ratio (95% CI)	no. of event / patient-yr	hazard ratio (95% CI)
**Major kidney events[b]**						
<80 mg/dl	40 / 1710	2.51 (1.82–3.46)	43 / 4269	1.75 (1.28–2.40)	105 / 1066	1.48 (1.20–1.81)
80–<100 mg/dl	236 / 18171	1.47 (1.26–1.70)	307 / 45860	1.50 (1.30–1.73)	507 / 7801	1.19 (1.06–1.32)
100–<110 mg/dl	268 / 27298	1.09 (0.95–1.26)	267 / 56172	1.17 (1.01–1.35)	460 / 9129	1.00 (0.89–1.12)
110–<126 mg/dl	704 / 74919	1.00	504 / 119779	1.00	920 / 19753	1.00
126–<140 mg/dl	682 / 78141	0.88 (0.79–0.98)	391 / 90056	1.02 (0.89–1.16)	734 / 17729	0.91 (0.83–1.01)
140–<160 mg/dl	985 / 97914	0.96 (0.87–1.06)	447 / 83443	1.16 (1.02–1.31)	818 / 19412	0.91 (0.83–1.01)
160–<180 mg/dl	923 / 68313	1.24 (1.12–1.37)	267 / 43479	1.26 (1.09–1.47)	602 / 12388	1.03 (0.93–1.15)
≥180 mg/dl	1916 / 100939	1.72 (1.57–1.88)	456 / 49802	1.89 (1.66–2.16)	1220 / 18840	1.32 (1.21–1.44)
**Doubling serum creatinine**						
<80 mg/dl	31 / 1570	2.24 (1.56–3.22)	18 / 3941	1.25 (0.78–2.02)	60 / 1172	1.08 (0.83–1.42)
80–<100 mg/dl	200 / 16100	1.45 (1.23–1.70)	171 / 39054	1.41 (1.17–1.71)	298 / 7290	1.12 (0.97–1.29)
100–<110 mg/dl	224 / 23820	1.06 (0.91–1.24)	159 / 47068	1.13 (0.93–1.37)	276 / 7959	1.03 (0.89–1.19)
110–<126 mg/dl	611 / 65322	1.00	313 / 99632	1.00	559 / 17278	1.00
126–<140 mg/dl	584 / 67631	0.87 (0.78–0.98)	245 / 74604	1.01 (0.85–1.19)	456 / 15193	0.93 (0.82–1.05)
140–<160 mg/dl	877 / 84442	0.99 (0.89–1.10)	275 / 68917	1.14 (0.97–1.34)	516 / 16746	0.91 (0.81–1.03)
160–<180 mg/dl	806 / 59085	1.26 (1.13–1.40)	168 / 35621	1.29 (1.07–1.56)	371 / 10918	0.99 (0.86–1.13)
≥180 mg/dl	1533 / 88254	1.62 (1.47–1.79)	277 / 41448	1.83 (1.55–2.15)	733 / 17245	1.22 (1.09–1.36)
**Kidney failure[c]**						
<80 mg/dl	17 / 1751	2.25 (1.38–3.68)	37 / 4288	2.03 (1.44–2.87)	85 / 1096	1.29 (1.03–1.62)
80–<100 mg/dl	104 / 18614	1.46 (1.17–1.82)	228 / 46176	1.56 (1.32–1.85)	459 / 8017	1.18 (1.05–1.32)
100–<110 mg/dl	122 / 27979	1.10 (0.89–1.36)	183 / 56503	1.18 (0.98–1.41)	427 / 9451	1.01 (0.90–1.14)
110–<126 mg/dl	330 / 76631	1.00	343 / 120508	1.00	838 / 20410	1.00
126–<140 mg/dl	315 / 79878	0.86 (0.73–1.00)	262 / 90652	1.01 (0.86–1.19)	642 / 18255	0.90 (0.81–1.00)
140–<160 mg/dl	427 / 100308	0.85 (0.74–0.98)	288 / 84009	1.08 (0.92–1.27)	721 / 19931	0.91 (0.82–1.00)
160–<180 mg/dl	427 / 70108	1.16 (1.01–1.35)	175 / 43808	1.18 (0.98–1.42)	539 / 12774	1.05 (0.94–1.17)
≥180 mg/dl	979 / 103915	1.79 (1.57–2.03)	336 / 50125	2.06 (1.76–2.40)	1022 / 19361	1.26 (1.14–1.38)
**Major cardiovascular events**						
<80 mg/dl	20 / 1777	1.73 (1.10–2.71)	41 / 4322	1.29 (0.94–1.77)	33 / 1328	1.87 (1.30–2.70)
80–<100 mg/dl	134 / 18675	1.14 (0.94–1.39)	377 / 46119	1.21 (1.06–1.37)	135 / 9301	1.11 (0.90–1.37)
100–<110 mg/dl	174 / 27944	1.03 (0.86–1.23)	402 / 56185	1.10 (0.97–1.24)	144 / 10600	1.06 (0.86–1.29)
110–<126 mg/dl	448 / 76338	1.00	777 / 119641	1.00	279 / 22564	1.00
126–<140 mg/dl	464 / 79452	1.00 (0.88–1.14)	595 / 90081	1.03 (0.92–1.15)	283 / 19891	1.15 (0.98–1.36)
140–<160 mg/dl	675 / 99875	1.17 (1.04–1.32)	678 / 83373	1.27 (1.14–1.41)	308 / 21803	1.14 (0.97–1.35)
160–<180 mg/dl	493 / 70027	1.23 (1.08–1.41)	391 / 43403	1.42 (1.26–1.61)	214 / 14296	1.22 (1.02–1.46)
≥180 mg/dl	928 / 104346	1.62 (1.44–1.82)	590 / 49990	1.89 (1.70–2.11)	409 / 22008	1.55 (1.32–1.81)
**All-cause mortality**						
<80 mg/dl	55 / 1797	1.96 (1.49–2.58)	150 / 4367	1.92 (1.62–2.27)	68 / 1365	1.38 (1.08–1.78)
80–<100 mg/dl	379 / 18879	1.33 (1.19–1.50)	1046 / 46701	1.26 (1.17–1.36)	397 / 9512	1.16 (1.02–1.31)
100–<110 mg/dl	448 / 28251	1.08 (0.97–1.20)	1058 / 56851	1.09 (1.01–1.17)	392 / 10822	1.02 (0.90–1.15)
110–<126 mg/dl	1097 / 77337	1.00	2054 / 121143	1.00	784 / 23054	1.00
126–<140 mg/dl	1038 / 80527	0.94 (0.86–1.02)	1621 / 91237	1.07 (1.00–1.14)	680 / 20359	1.01 (0.91–1.12)
140–<160 mg/dl	1419 / 101315	1.05 (0.97–1.14)	1571 / 84596	1.14 (1.07–1.22)	712 / 22355	0.98 (0.89–1.09)

© 2021 Jung HH et al. *JAMA Network Open.*

160–<180 mg/dl	1071 / 71020	1.20 (1.10–1.31)	938 / 44161	1.34 (1.24–1.45)	526 / 14613	1.19 (1.06–1.33)
≥180 mg/dl	2049 / 106092	1.68 (1.56–1.81)	1490 / 50918	1.94 (1.82–2.08)	1012 / 22616	1.57 (1.42–1.72)

[a] The hazard ratios were adjusted for time-varying covariates of antidiabetic use status and untreated FBG and baseline covariates of age, sex, eGFR, proteinuria, time of antihypertensive initiation, antihypertensive and statin use statuses, fasting glucose, total cholesterol, HDL cholesterol, LDL cholesterol, body mass index, family history of cardiovascular disease, income, smoking, physical exercise, and drinking.
[b] Major kidney event was a composite of kidney failure or doubling of serum creatinine.
[c] Kidney failure was identified as dialysis for ≥90 days per year, kidney transplantation, or death from CKD.
FBG, fasting blood glucose; CKD, chronic kidney disease.

© 2021 Jung HH et al. *JAMA Network Open.*

eTable 7. Hazard Ratios of On-Treatment Fasting Blood Glucose Level Among Participants With No Chronic Kidney Disease[a]

**Outcome**	**No CKD**	
FBG	no. of event / patient-yr	hazard ratio (95% CI)
**Major kidney events[b]**		
<80 mg/dl	11 / 2218	1.67 (0.91–3.07)
80–<100 mg/dl	94 / 28073	1.29 (1.01–1.65)
100–<110 mg/dl	100 / 40439	1.01 (0.80–1.27)
110–<126 mg/dl	239 / 99356	1.00
126–<140 mg/dl	259 / 86597	1.23 (1.03–1.47)
140–<160 mg/dl	239 / 85849	1.15 (0.96–1.38)
160–<180 mg/dl	164 / 46695	1.45 (1.19–1.78)
≥180 mg/dl	315 / 55571	2.31 (1.93–2.75)
**Doubling serum creatinine**		
<80 mg/dl	10 / 2073	1.42 (0.75–2.69)
80–<100 mg/dl	91 / 25004	1.27 (1.00–1.63)
100–<110 mg/dl	95 / 35667	0.98 (0.77–1.25)
110–<126 mg/dl	230 / 86859	1.00
126–<140 mg/dl	251 / 75252	1.24 (1.04–1.49)
140–<160 mg/dl	231 / 74382	1.15 (0.96–1.39)
160–<180 mg/dl	153 / 40309	1.44 (1.17–1.77)
≥180 mg/dl	276 / 48077	2.20 (1.83–2.64)
**Kidney failure[c]**		
<80 mg/dl	1 / 2223	2.04 (0.26–15.69)
80–<100 mg/dl	8 / 28371	1.39 (0.61–3.16)
100–<110 mg/dl	7 / 40847	0.82 (0.35–1.93)
110–<126 mg/dl	22 / 100338	1.00
126–<140 mg/dl	18 / 87590	0.88 (0.47–1.64)
140–<160 mg/dl	24 / 86916	1.07 (0.60–1.91)
160–<180 mg/dl	32 / 47281	2.42 (1.40–4.20)
≥180 mg/dl	86 / 56347	5.10 (3.13–8.31)
**Major cardiovascular events**		
<80 mg/dl	8 / 2213	1.08 (0.53–2.18)
80–<100 mg/dl	118 / 28188	1.27 (1.03–1.58)
100–<110 mg/dl	161 / 40568	1.21 (1.00–1.46)
110–<126 mg/dl	324 / 99570	1.00
126–<140 mg/dl	297 / 86913	1.06 (0.91–1.24)
140–<160 mg/dl	334 / 86208	1.23 (1.05–1.43)
160–<180 mg/dl	232 / 46821	1.62 (1.37–1.93)
≥180 mg/dl	324 / 55826	2.04 (1.74–2.40)
**All-cause mortality**		
<80 mg/dl	50 / 2225	2.38 (1.78–3.17)
80–<100 mg/dl	336 / 28394	1.32 (1.16–1.50)
100–<110 mg/dl	417 / 40856	1.16 (1.03–1.31)
110–<126 mg/dl	861 / 100363	1.00
126–<140 mg/dl	723 / 87635	0.98 (0.89–1.09)
140–<160 mg/dl	774 / 86966	1.10 (1.00–1.21)
160–<180 mg/dl	483 / 47349	1.32 (1.18–1.48)
≥180 mg/dl	724 / 56481	1.85 (1.67–2.05)

[a] The hazard ratios were adjusted for time-varying covariates of antidiabetic use status and untreated FBG and baseline covariates of age, sex, eGFR, proteinuria, time of antihypertensive initiation, antihypertensive and statin use statuses, fasting glucose, total cholesterol, HDL cholesterol, LDL cholesterol, body mass index, family history of cardiovascular disease, income, smoking, physical exercise, and drinking.
[b] Major kidney event was a composite of kidney failure or doubling of serum creatinine.
[c] Kidney failure was identified as dialysis for ≥90 days per year, kidney transplantation, or death from CKD.
FBG, fasting blood glucose; CKD, chronic kidney disease.

© 2021 Jung HH et al. *JAMA Network Open.*

eTable 8. Time-Dependent Interactions of On-Treatment Fasting Blood Glucose Level and a Log Function of Follow-Up Time

Outcome	CKD with Dipstick Albuminuria		CKD with eGFR <60 ml/min/1.73 m^2		General Population	
FBG	Wald chi-square	P for interaction	Wald chi-square	P for interaction	Wald chi-square	P for interaction
**Major kidney event[a]**						
<80 mg/dl	0.438	0.508	0.894	0.344	0.370	0.543
80–<100 mg/dl	1.815	0.178	4.364	0.037	0.530	0.467
100–<110 mg/dl	0.814	0.367	1.497	0.221	0.455	0.500
110–<126 mg/dl[b]	.	.	.	.	.	.
126–<140 mg/dl	0.901	0.342	0.526	0.468	0.654	0.419
140–<160 mg/dl	1.938	0.164	0.852	0.356	0.266	0.606
160–<180 mg/dl	2.439	0.118	1.073	0.300	4.359	0.037
≥180 mg/dl	9.954	0.002	1.310	0.252	18.773	<.0001
**All-cause mortality**						
<80 mg/dl	0.134	0.715	1.457	0.227	0.540	0.462
80–<100 mg/dl	2.419	0.120	1.959	0.162	4.413	0.036
100–<110 mg/dl	3.374	0.066	2.157	0.142	2.259	0.133
110–<126 mg/dl[b]	.	.	.	.	.	.
126–<140 mg/dl	3.900	0.048	2.128	0.145	9.646	0.002
140–<160 mg/dl	0.567	0.452	0.773	0.379	0.085	0.771
160–<180 mg/dl	0.501	0.479	0.003	0.957	0.273	0.602
≥180 mg/dl	0.000	0.984	0.931	0.335	2.981	0.084

[a] Major kidney event was a composite of doubling of serum creatinine, end-stage kidney disease, or death from CKD.
[b] The on-treatment FBG 110 to <126 mg/dl was set as the reference.
CKD, chronic kidney disease; eGFR, estimated glomerular filtration rate; FBG, fasting blood glucose.

© 2021 Jung HH et al. *JAMA Network Open.*

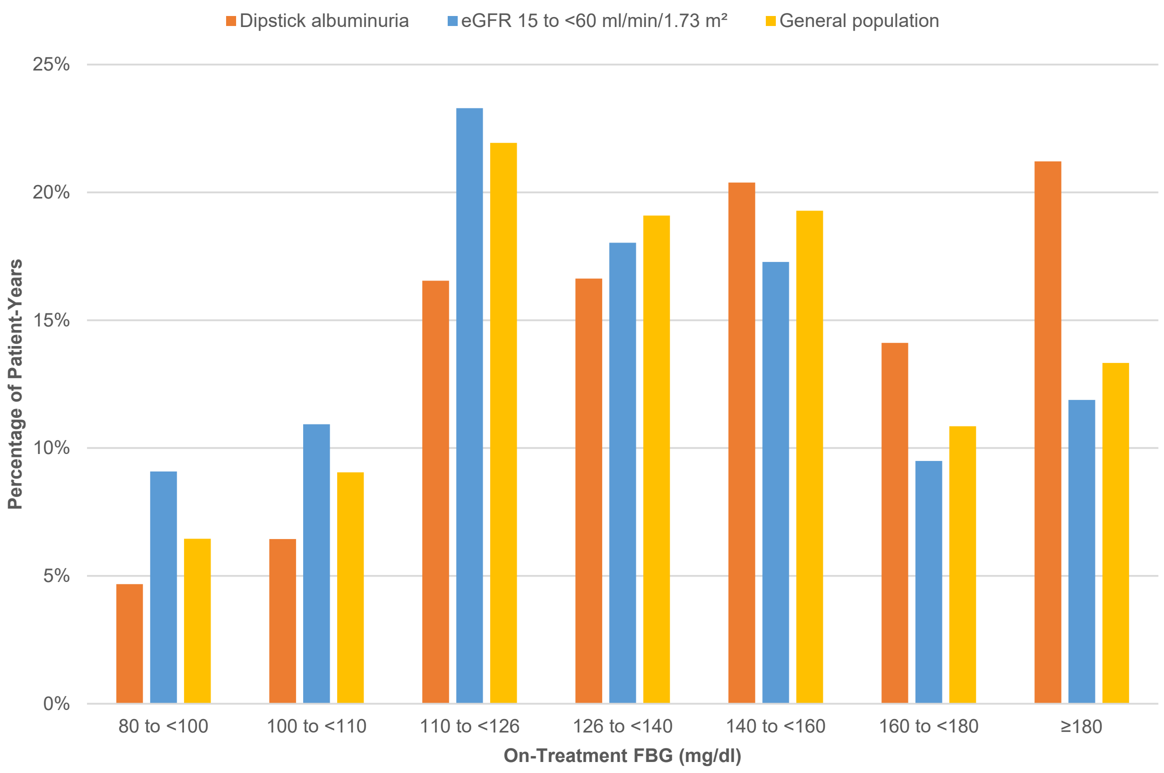

eFigure 1. Distribution of Follow-Up Fasting Blood Glucose Level in Albuminuria, Decreased Estimated Glomerular Filtration Rate, and General Populations

The percentages of patient-years (Y-axis) across on-treatment FBG categories (X-axis) were calculated in each of the albuminuria, decreased eGFR, and general populations. The rare FBG <80 mg/dl is excluded from the plots. eGFR, estimated glomerular filtration rate; FBG, fasting blood glucose.

© 2021 Jung HH et al. *JAMA Network Open.*

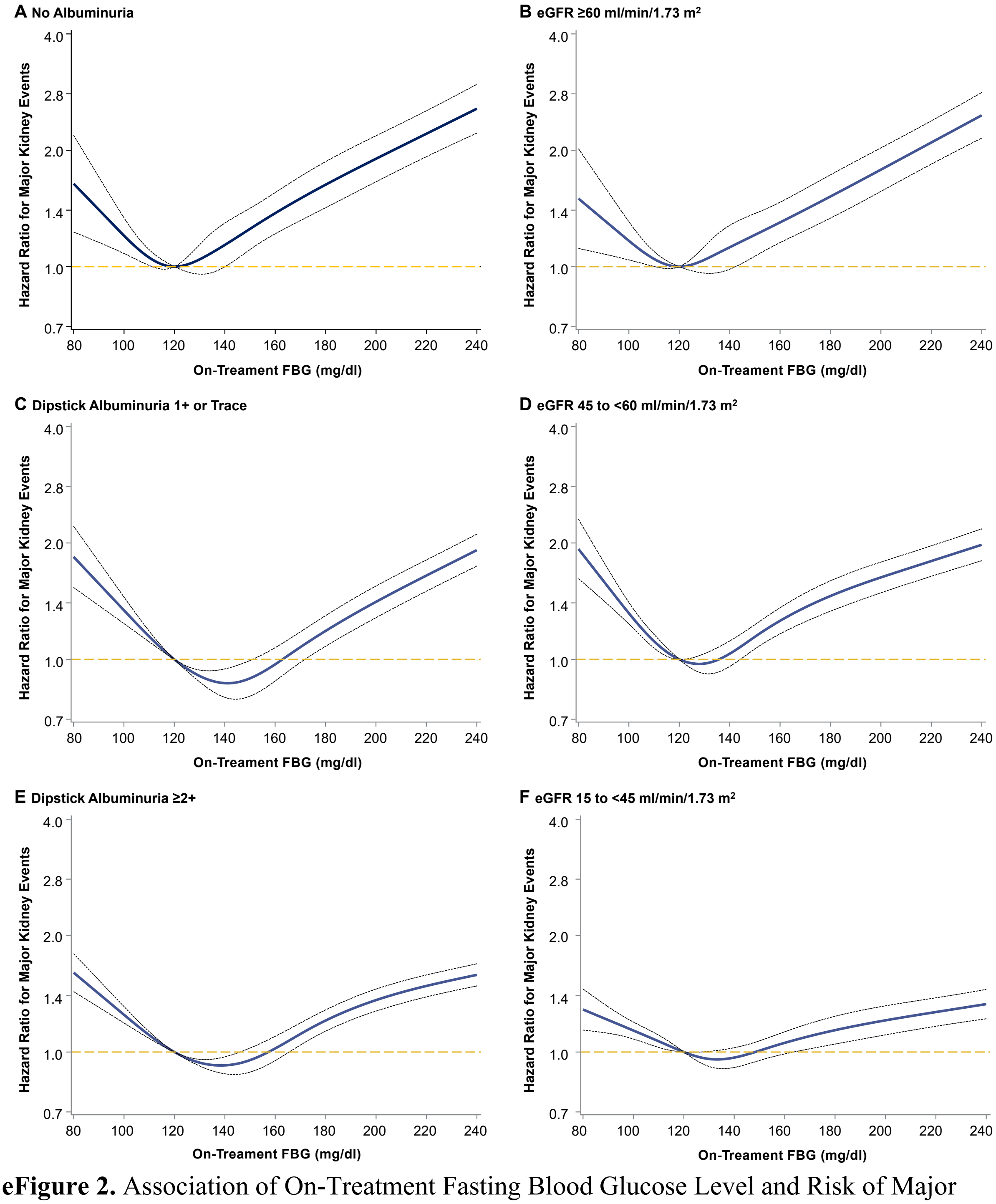

eFigure 2. Association of On-Treatment Fasting Blood Glucose Level and Risk of Major Kidney Events by Albuminuria or Estimated Glomerular Filtration Rate Level

The hazard ratios (solid line) and 95% CIs (dotted line) were estimated using a restricted cubic spline function. The on-treatment FBG 120 mg/dl was set as the reference (dashed line). The major kidney event was a composite of doubling serum creatinine, end-stage kidney disease, or death from chronic kidney disease. Analyses were adjusted for time-varying covariates of antidiabetic use status and untreated FBG and fixed covariates of baseline age, sex, eGFR, proteinuria, time of antidiabetic initiation, antihypertensive and statin use statuses, systolic blood pressure, total cholesterol, HDL cholesterol, LDL cholesterol, body mass index, family history of cardiovascular disease, income, smoking, physical exercise, and drinking. eGFR, estimated glomerular filtration rate; FBG, fasting blood glucose.

© 2021 Jung HH et al. *JAMA Network Open.*

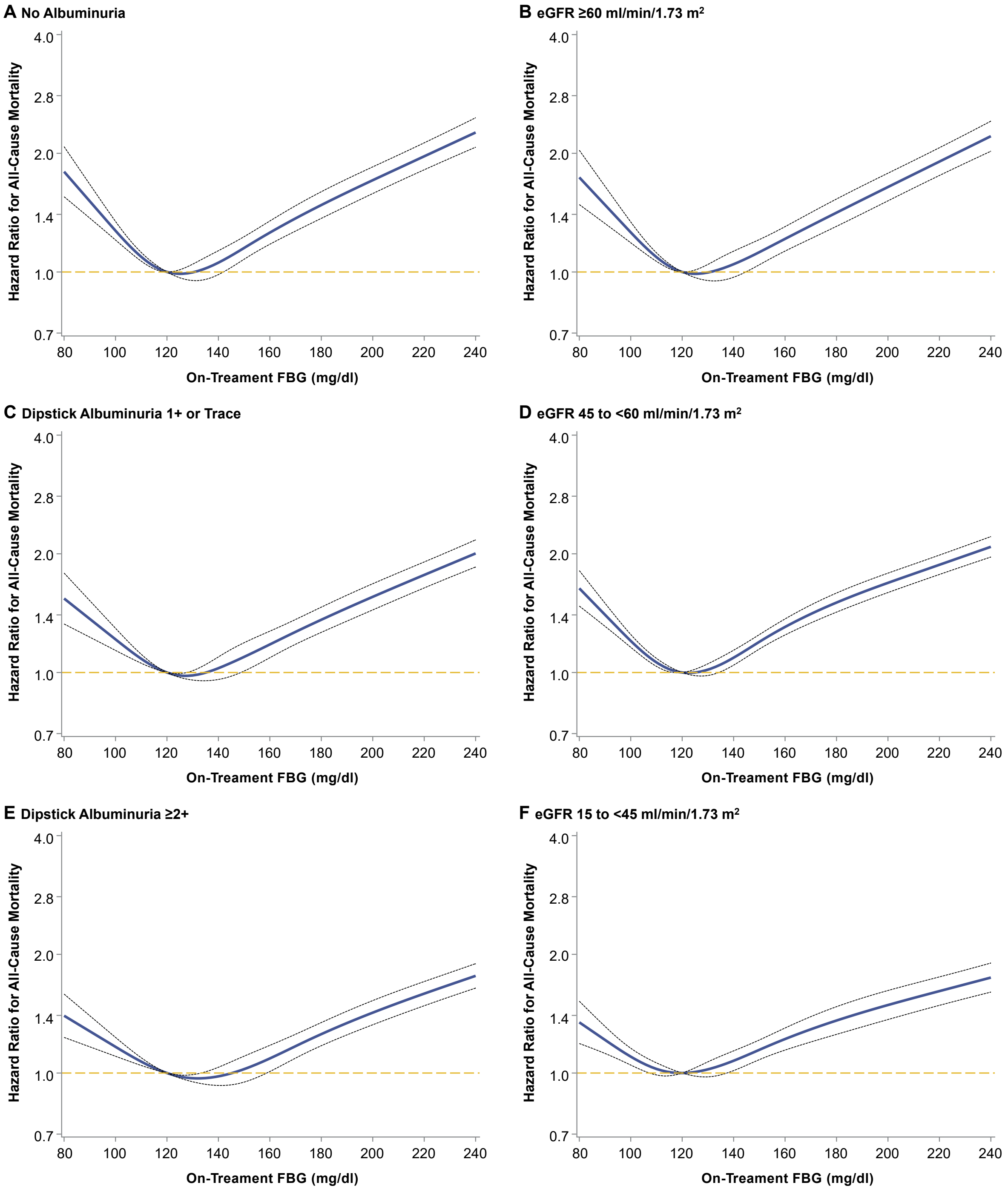

eFigure 3. Association of On-Treatment Fasting Blood Glucose Level and All-Cause Mortality by Albuminuria or Estimated Glomerular Filtration Rate Level

The hazard ratios (solid line) and 95% CIs (dotted line) were estimated using a restricted cubic spline function. The on-treatment FBG 120 mg/dl was set as the reference (dashed line). Analyses were adjusted for time-varying covariates of antidiabetic use status and untreated FBG and fixed covariates of baseline age, sex, eGFR, proteinuria, time of antidiabetic initiation, antihypertensive and statin use statuses, systolic blood pressure, total cholesterol, HDL cholesterol, LDL cholesterol, body mass index, family history of cardiovascular disease, income, smoking, physical exercise, and drinking. eGFR, estimated glomerular filtration rate; FBG, fasting blood glucose.

© 2021 Jung HH et al. *JAMA Network Open.*

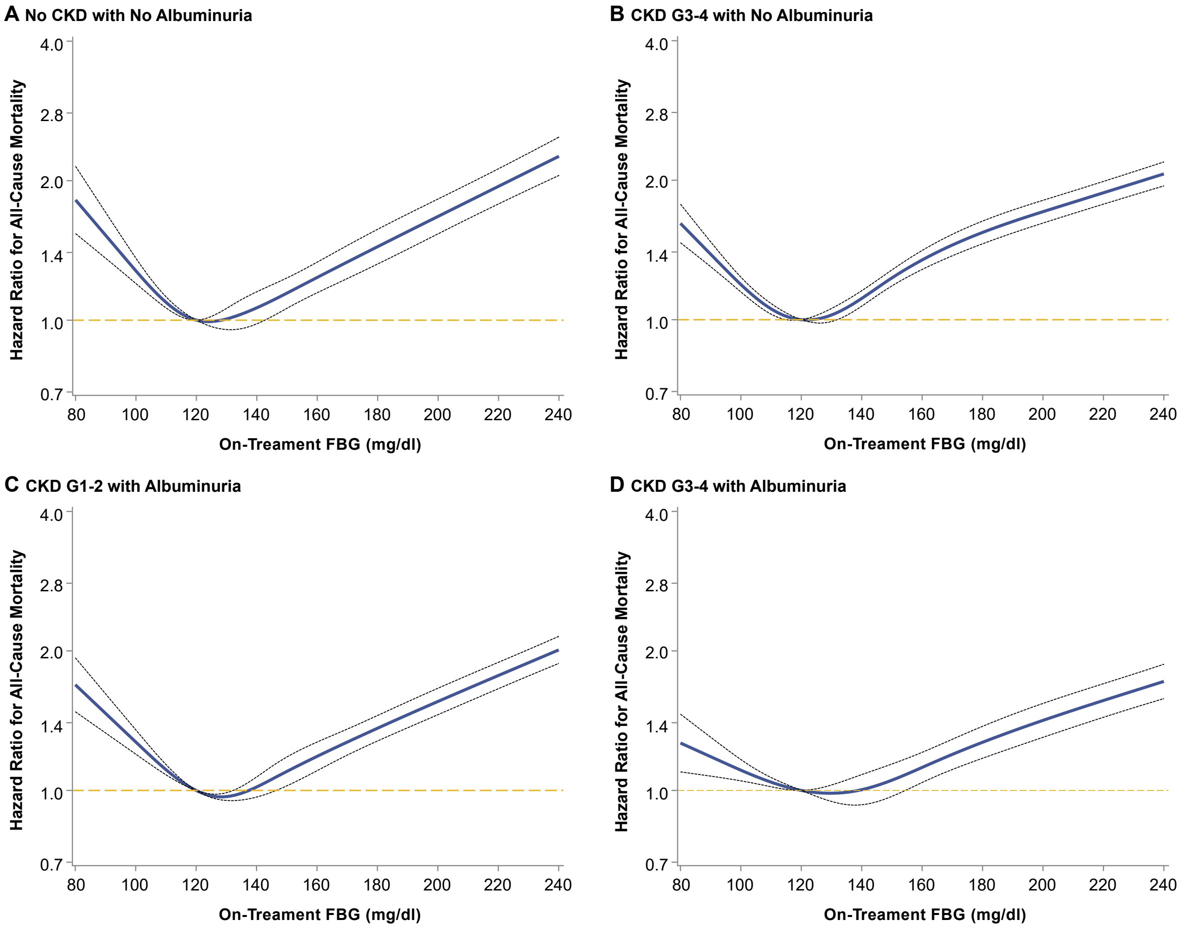

eFigure 4. Association of On-Treatment Fasting Blood Glucose Level and All-Cause Mortality by Presence or Absence of Albuminuria and Decreased Estimated Glomerular Filtration Rate

The hazard ratios (solid line) and 95% CIs (dotted line) were estimated using a restricted cubic spline function. The on-treatment FBG 120 mg/dl was set as the reference (dashed line). Analyses were adjusted for time-varying covariates of antidiabetic use status and untreated FBG and fixed covariates of baseline age, sex, eGFR, proteinuria, time of antidiabetic initiation, antihypertensive and statin use statuses, systolic blood pressure, total cholesterol, HDL cholesterol, LDL cholesterol, body mass index, family history of cardiovascular disease, income, smoking, physical exercise, and drinking. CKD G1-2 and CKD G3-4 denote CKD with an eGFR ≥60 ml/min/1.73 m^2 and CKD with an eGFR 15 to <60 ml/min/1.73 m2, respectively. CKD, chronic kidney disease; FBG, fasting blood glucose.

© 2021 Jung HH et al. *JAMA Network Open.*

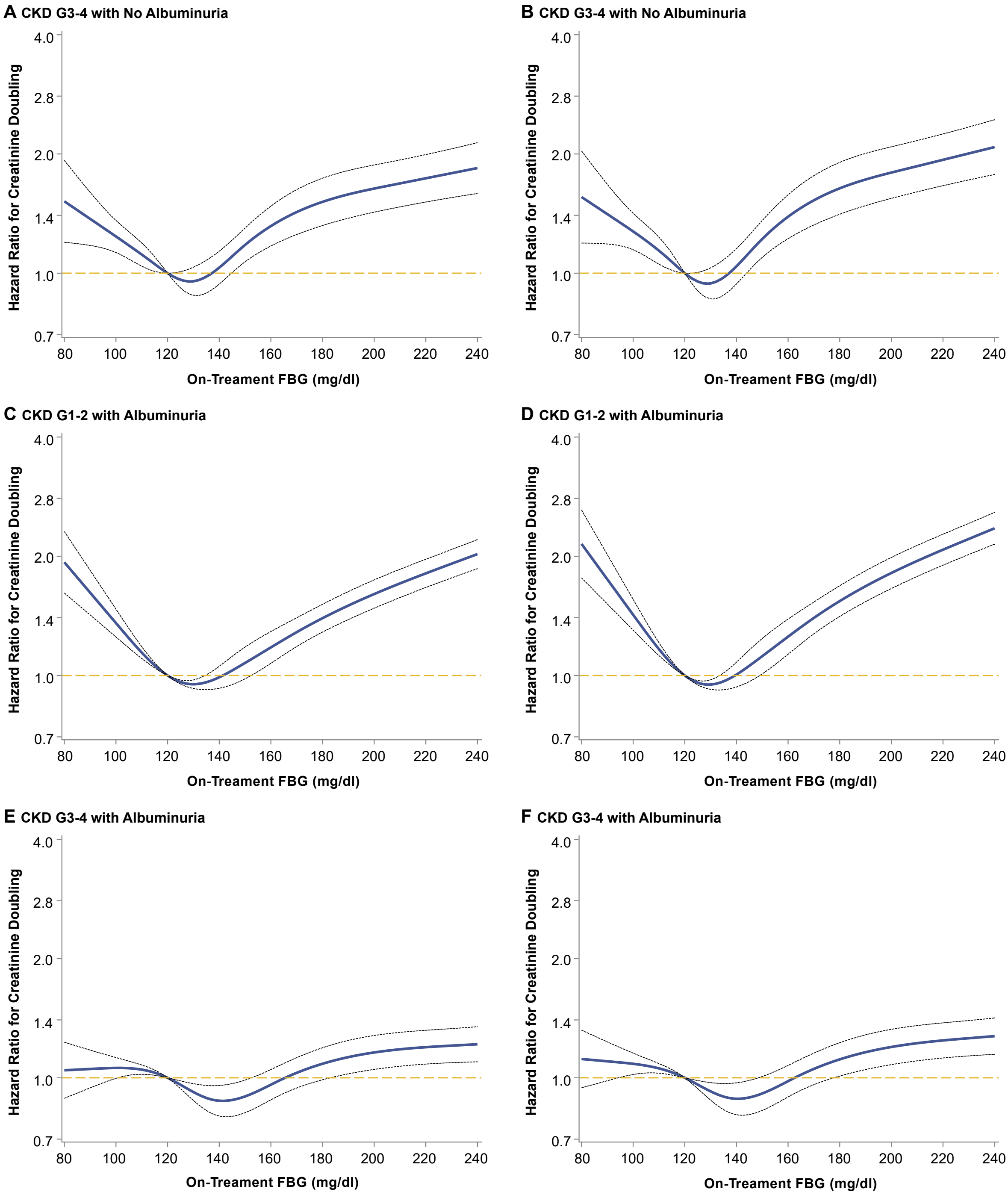

eFigure 5. Association of On-Treatment Fasting Blood Glucose Level and Risk of Doubling Serum Creatinine Before and After Exclusion of Transient Creatinine Doubling

The hazard ratios (solid line) and 95% CIs (dotted line) were estimated using a restricted cubic spline function. The on-treatment FBG 120 mg/dl was set as the reference (dashed line). Analyses were adjusted for time-varying covariates of antidiabetic use status and untreated FBG and fixed covariates of baseline age, sex, eGFR, proteinuria, time of antidiabetic initiation, antihypertensive and statin use statuses, systolic blood pressure, total cholesterol, HDL cholesterol, LDL cholesterol, body mass index, family history of cardiovascular disease, income, smoking, physical exercise, and drinking. CKD, chronic kidney disease; FBG, fasting blood glucose.

© 2021 Jung HH et al. *JAMA Network Open.*

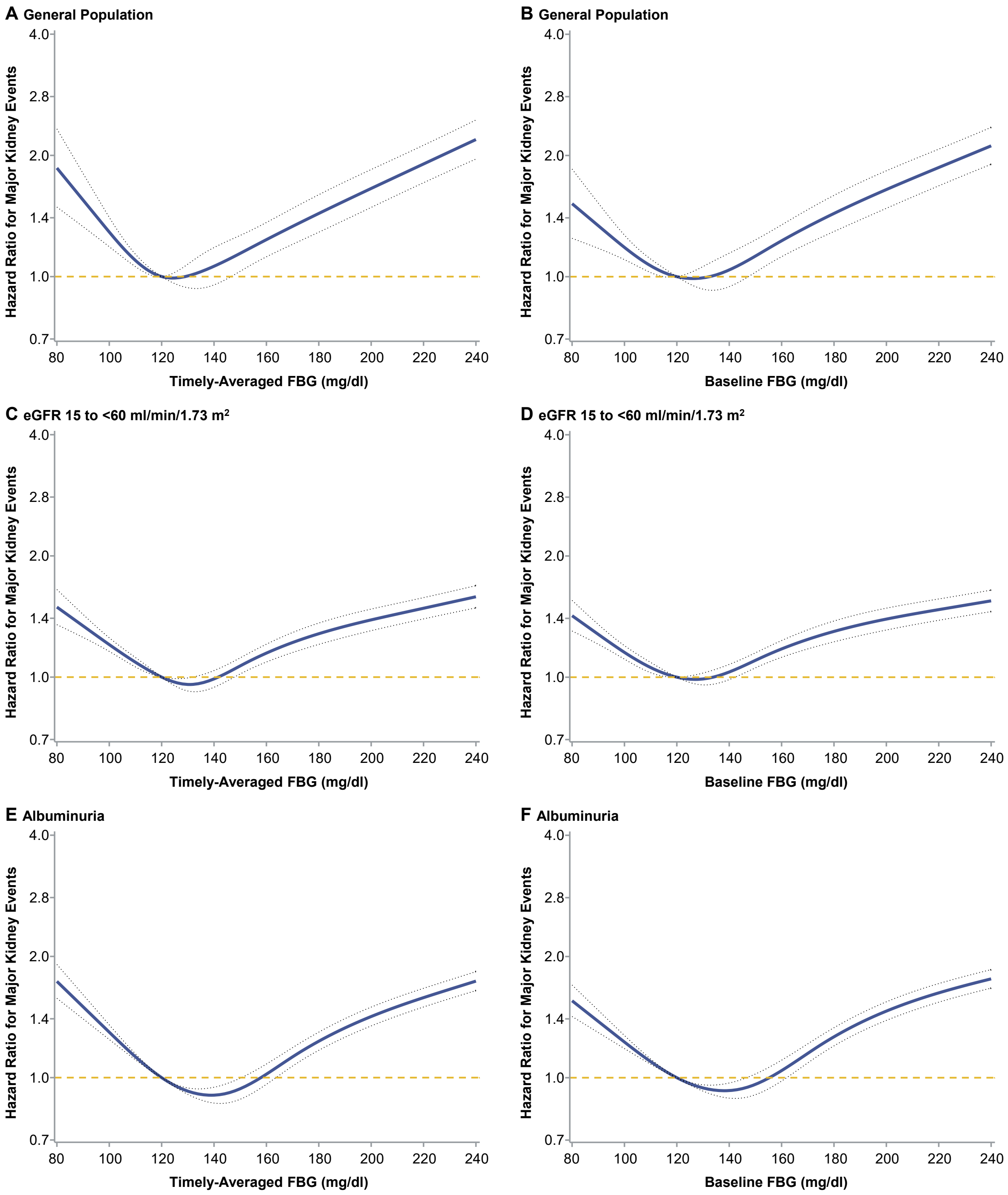

eFigure 6. Comparison of Kidney Risks of Time-Updated and Baseline Fasting Blood Glucose Level

The hazard ratios (solid line) and 95% CIs (dotted line) were estimated using a restricted cubic spline function. The on-treatment FBG 120 mg/dl was set as the reference (dashed line). The major kidney event was a composite of doubling serum creatinine, end-stage kidney disease, or death from chronic kidney disease. Analyses were adjusted for time-varying covariates of antidiabetic use status and untreated FBG and fixed covariates of baseline age, sex, eGFR, proteinuria, time of antidiabetic initiation, antihypertensive and statin use statuses, systolic blood pressure, total cholesterol, HDL cholesterol, LDL cholesterol, body mass index, family history of cardiovascular disease, income, smoking, physical exercise, and drinking. eGFR, estimated glomerular filtration rate; FBG, fasting blood glucose.

© 2021 Jung HH et al. *JAMA Network Open.*

eReferences.

1. Yadlowsky S, Hayward RA, Sussman JB, McClelland RL, Min Y-I, Basu S. Clinical Implications of Revised Pooled Cohort Equations for Estimating Atherosclerotic Cardiovascular Disease Risk. *Ann Intern Med*. 2018;169(1):20-29. doi:10.7326/M17-3011
2. Expert Panel on Detection E and Treatment of High Blood Cholesterol in Adults. Executive Summary of The Third Report of The National Cholesterol Education Program (NCEP) Expert Panel on Detection, Evaluation, And Treatment of High Blood Cholesterol In Adults (Adult Treatment Panel III). *JAMA*. 2001;285(19):2486-2497.
3. WHO Expert Consultation. Appropriate body-mass index for Asian populations and its implications for policy and intervention strategies. *Lancet*. 2004;363(9403):157-163. doi:10.1016/S0140-6736(03)15268-3
4. Alcohol Facts and Statistics. National Institute on Alcohol Abuse and Alcoholism (NIAAA). Published April 25, 2019. Accessed November 9, 2019. https://www.niaaa.nih.gov/publications/brochures-and-fact-sheets/alcohol-facts-and-statistics

© 2021 Jung HH et al. *JAMA Network Open.*
